# Bilateral intermittent theta burst stimulation over the primary motor cortex improves motor and affective symptoms via thalamic network reintegration in mid-stage Parkinson's disease

**DOI:** 10.1016/j.neurot.2026.e00911

**Published:** 2026-04-28

**Authors:** Paloma Macías-García, Raúl Rashid-López, Álvaro J. Cruz-Gómez, Francisco L. Sánchez-Fernández, Esteban Sarrias-Arrabal, Fátima Cano-Cano, Elena Lozano-Soto, Florencia Sanmartino, Raúl Espinosa-Rosso, Javier J. González-Rosa

**Affiliations:** a Department of Psychology, University of Cadiz, Cadiz, Spain; b Institute of Research and Innovation in Biomedical Sciences of Cadiz (INiBICA), Cadiz, Spain; c Department of Neurology, Puerta del Mar University Hospital, Cadiz, Spain

**Keywords:** Functional connectivity, Intermittent theta burst stimulation, Parkinson's disease, Motor and nonmotor symptoms, Resting-state functional magnetic neuroimaging

## Abstract

Bilateral intermittent theta burst stimulation (iTBS) targeting the primary motor cortex (M1) has emerged as a promising neuromodulatory approach for Parkinson's disease (PD), although evidence supporting its clinical efficacy as an adjunctive treatment remains limited. This randomized, double-blind, sham-controlled crossover study examined the therapeutic potential of bilateral M1-iTBS in fifteen patients with mid-stage PD in the on-medication state. Motor, neuropsychiatric, and cognitive outcomes were assessed through standardized clinical scales following five iTBS sessions, alongside voxelwise resting-state functional magnetic resonance imaging, to identify neural-related mechanisms. Real iTBS induced a maximum improvement of nine points on the Movement Disorder Society-Unified PD Rating Scale part III, with clinical responders (≥20% improvement) demonstrating an eleven-point enhancement. Responders exhibited strengthened cerebello-thalamic and thalamo-cortical connectivity, suggesting partial thalamic functional reintegration with associative cortices and compensatory functional reorganization, which correlated significantly with reduced bradykinesia. Additionally, real iTBS produced clinically meaningful improvement in affective symptomatology in depression and anxiety responder patients, accompanied by modulation of top-down and bottom-up emotion regulation circuits and normalization of cerebellar activity. These findings support bilateral M1-iTBS as an effective adjunctive intervention for managing motor and nonmotor manifestations in patients with mid-stage PD, with mechanistic insights provided by symptom-related functional connectivity changes.

## Introduction

Parkinson's disease (PD), traditionally defined as a motor disorder due to the manifestation of bradykinesia, tremor, rigidity and postural instability [1,2], should instead be conceptualized as a multisystem disease, as it is also characterized by a heterogeneous range of nonmotor symptoms, such as depression, anxiety and apathy, alongside a wide spectrum of autonomic and sensory disturbances [[3], [4], [5]]. Neurodegeneration of dopaminergic neurons within the nigrostriatal pathway has been traditionally linked to dysfunction of cortico-striatal-thalamic circuits, accounting for both motor and nonmotor symptoms in PD, although not exclusively [6].

Dopamine drug replacement therapy, principally represented by levodopa, is the cornerstone of PD symptom management. However, owing to its limited half-life effect and the emergence of drug-induced dyskinesias in more advanced stages, combined with motor and nonmotor symptom fluctuations, the development of adjunctive therapies has become a clinical priority [7]. Repetitive transcranial magnetic stimulation (rTMS) and intermittent theta burst stimulation (iTBS) are two excitatory noninvasive brain stimulation methods that have yielded promising results in ameliorating motor [[8], [9], [10]] and nonmotor symptoms [11,12] in patients with PD. These protocols likely enhance adaptive functional activity patterns, attenuate maladaptive dynamics, and restore neural homeostasis within imbalanced networks [13].

The depiction of the brain as a set of large-scale intrinsically organized regions within and between dynamic network connections permits rTMS-induced activity to propagate across interconnected circuits, thereby exerting anatomical, functional and neurophysiological modulation [14,15]. Resting-state fMRI (rs-fMRI) is sensitive to the identification of nonfocal interactions, making it particularly suited for detecting transcranial magnetic stimulation (TMS)-induced effects that propagate beyond the stimulated region at voxel-level resolution. Functional connectivity (FC) alterations in patients with PD have been documented in motor cortices, parietal associative areas and cerebellum following excitatory and inhibitory rTMS protocols applied to primary motor cortex (M1), premotor cortex, and supplementary motor areas [16]. Nevertheless, a recent meta-analysis revealed that although positive results have been reported from rs-fMRI and rTMS studies, robust conclusions are limited by variability in study population, stimulation parameters, and FC analytical methods [17]. Most iTBS studies in PD have focused on clinical outcomes or cortical excitability, leaving the network-level mechanisms of stimulation insufficiently characterized. Although rs-fMRI offers a powerful approach to examine such large-scale functional alterations, it has rarely been combined with iTBS in this population [18]. The present study addresses this gap by jointly evaluating motor and mood-related effects together with voxelwise rs-fMRI connectivity, leveraging the extensive motor–limbic projections of the primary motor cortex as a mechanistic basis for bilateral stimulation. By integrating clinical and neuroimaging measures within a randomized double-blind crossover design, our work enables a comprehensive evaluation of both clinical effects and their underlying neural correlates following bilateral M1-iTBS.

Given the clinical evidence and this mechanistic framework, we adopted a multimodal approach combining bilateral iTBS targeting M1 with subsequent rs-fMRI acquisition to assess motor and mood-related nonmotor symptoms in patients with mid-stage PD. M1 offers both direct and indirect projections to diverse cortico-subcortical regions via BG, which acts as an intermediary hub within motor, mesocortical, and limbic pathways [19,20]. Beyond its role in motor execution, the M1 is tightly interconnected with cortico-striatal and cortico-limbic circuits that are disrupted in PD, linking M1 activity to both motor and affective manifestations [21]. Neuroimaging studies have shown that M1 participates in cognitive-affective processes in PD and that motor disturbances may arise from large-scale motor and non-motor dysfunctional interaction rather than isolated regional alterations [21,22]. Moreover, neuromodulation of M1 has been associated with mood improvements in several neurological and chronic pain conditions [23,24], suggesting that M1 stimulation may influence affective symptoms in PD through shared dysfunctional networks [21,22]. This network-based perspective supports the rationale for assessing whether bilateral M1-iTBS can modulate both motor and mood-related symptoms in PD. Therefore, the possibility of simultaneously modulating interconnected limbic circuits, and thereby improving mood-related symptoms, represents a meaningful advantage. On the basis of these anatomical considerations and the potential network-level effects of iTBS, we hypothesized that bilateral M1-iTBS would concurrently improve motor and mood-related symptoms in patients with mid-stage PD through large-scale functional reorganization.

## Materials and Methods

### Study design

We performed a single-centre, randomized, double-blind, sham-controlled crossover trial (Supplementary Fig. 1) in which patients were randomly assigned (1:1) to receive either real or sham/placebo iTBS (Phase I). Following a minimum three-month washout period, patients were crossed over to the opposite treatment condition (Phase II). A minimum 3-month washout period was determined conservatively to minimize potential residual carry-over effects from the first intervention phase. This interval was chosen based on prior rTMS and TBS crossover studies in PD [25] showing that stimulation-induced neuroplastic changes may dissipate within several weeks and that washout intervals longer than one month are recommended. Both Phase I and Phase II followed an identical protocol, consisting of (i) baseline evaluation, (ii) neuronavigated real or sham iTBS over nonsimultaneous bilateral M1 (one daily session for five consecutive days), and (iii) posttreatment follow-up assessment. Ethical approval for the study was granted by the Andalusian Biomedical Research Ethics Committee (reference no. 2169-N-19) on April 1, 2020, in compliance with the Declaration of Helsinki and subsequent amendments. All participants were naïve to TMS and provided written informed consent after receiving a full explanation of the study procedures prior to the enrollment.

Extended methods are detailed in the methodology section of our protocol paper [26], since the present study primarily provides some of the main analysis and variables from the principal study design.

Baseline and posttreatment follow-up evaluations consisted of (i) clinical motor assessment (the Movement Disorder Society - Unified Parkinson's Disease Rating Scale, MDS-UPDRS, parts II, III & IV), (ii) cognitive and neuropsychiatric assessment, and (iii) functional MRI (fMRI) scans.

### Sample size calculation

The sample size was determined through power analysis informed by previous rTMS-PD studies. For a 2 × 2 crossover design with 80% power to detect a clinically meaningful treatment effect (α = 0.05, two-tailed) and accounting for an anticipated 20% dropout rate, the initial target sample was established at 24 patients. Owing to recruitment constraints inherent to specialized neuromodulation trials, the final enrolled sample comprised 15 patients. Post hoc power analysis confirmed adequate statistical power for primary motor outcome measures.

### Demographic information and inclusion and exclusion criteria of participants

#### Inclusion criteria

Participants were eligible if they met the following conditions: (i) PD diagnosis according to the United Kingdom Parkinson's Disease Society Brain Bank diagnostic criteria (27); (ii) disease duration of at least five years; (iii) disease symptomatology in the ON medication state corresponding to Hoehn & Yahr (H&Y) scale of II-III; (iv) clinical and therapeutic stability for at least two months; and (v) age ranging from 45 to 75 years.

#### Exclusion criteria

Participants were excluded if they (i) manifested an important systemic disease; (ii) exhibited moderate or severe cognitive impairment as indicated by a Mini-Mental Parkinson's (MMP) score of ≤24; (iii) presented incapacitating psychiatric or clinical conditions, such as dystonia and/or dyskinesia, that may hinder the proper execution of the protocol; or (iv) had evidence of atypical parkinsonism, neurological comorbidities, a history of cranioencephalic trauma or epilepsy, or any other contraindication to TMS neurostimulation. Patients with moderate-to-severe cognitive impairment were excluded to ensure valid informed consent, reliable task and clinical assessment compliance, and feasibility of repeated MRI/TMS procedures, while also reducing confounding related to more advanced multisystem disease.

#### Cohort characteristics: demographic information

A total of 24 patients diagnosed with PD were initially recruited from the Movement Disorders unit (Neurology Department, Puerta del Mar University Hospital, Cádiz, Spain), in accordance with the United Kingdom Parkinson's Disease Society Brain Bank diagnostic criteria [27,28]. Nine patients were excluded because of pharmacological readjustment during the experiment or noncompletion of the second treatment phase in the crossover trial. The final sample consisted of 15 patients with PD (8 males; mean age: 58.1 ± 8.5 years; mean disease duration: 8.7 ± 3.7 years) (Table 1). The dosage of dopaminergic medication was maintained at a stable level for at least two months prior to the study and throughout the duration of the trial.

**Table 1:** Demographic information.

	Sex (M:F)	Age	Disease duration (years)	H&Y stage	LEDD (mg/day)	PD subtype	More affected hemibody	1st baseline		1st treatment (Sham:Real)
								MDS-UPDRS part III	MMP	
**Full sample**	8:7	58.10 ± 8.50	8.70 ± 3.72	2.30 ± 0.32	966.00 ± 289.52	PIGD 8; Mixed 1; TD 6	8:7	25.40 ± 8.81	29.20 ± 2.43	9:6
**MDS-UPDRS responders**	4:7	57.90 ± 7.06	8.90 ± 4.24	2.36 ± 0.32	902.45 ± 299.26	Mixed 1; PIGD 5; TD 5	5:6	23.36 ± 7.24	28.82 ± 2.68	5:6
**HDRS responders**	3:4	59.71 ± 8.50	9.14 ± 2.69	2.36 ± 0.24	979.00 ± 2.69	PIGD 5; TD 2	3:4	25.57 ± 12.49	29.00 ± 2.48	2:4
**HARS responders**	3:3	59.50 ± 10.67	8.08 ± 2.33	2.50 ± 0.32	1033.33 ± 228.64	PIGD 3; TD 3	4:2	27.00 ± 10.60	29.17 ± 2.48	2:4

**Abbreviations***:* F, Female; M, Male; H&Y, HDRS, Hamilton Depression Rating Scale; HARS, Hamilton Anxiety Rating Scale; Hoehn & Yahr scale; L, Left; R, Right; LEDD, Levodopa equivalent daily dose; MDS-UPDRS, Movement Disorder Society-Unified Parkinson's Disease Rating Scale; MMP, Mini Mental Parkinson; PD, Parkinson's disease; PIGD, Postural/Instability Gait difficulties; TD, Tremor dominant.

### Randomization, blinding procedures and integrity of the blind

Only the investigator responsible for randomization was aware of the patients’ group allocation. On the first stimulation session day, treatment conditions (either real or sham) were revealed to the investigator in charge of applying the iTBS protocol. Patients and evaluators remained blinded until the full completion of phase II.

To ensure data integrity, patients were asked which treatment condition– first real iTBS or sham iTBS – they had received first. A total of 2 participants from the first real iTBS group condition provided no answer to the question. Three out of eight subjects (38%) from this group correctly identified the received treatment. On the other hand, three out of seven (43%) subjects guessed the treatment order. No significant differences were observed between groups (χ^2^_(2, n=15)_ = 2.07, p = 0.35).

### Intermittent theta burst stimulation protocol

**Fig. 1:**
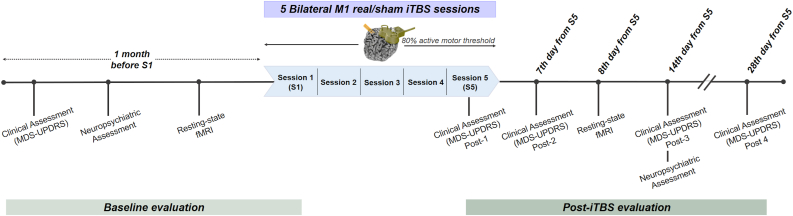
**Study pipeline of scheduled assessment time organization during Phase I and Phase II**. Patients underwent same scheduled assessments during Phase I and Phase II, which were identical. Phase I and Phase II differed only in the counterbalanced treatment condition (sham or real iTBS), whereby each participant received the opposite condition in Phase II. **Abbreviations:** S1–S5 refer to M1-iTBS session 1-session 5; MDS-UPDRS: The Movement Disorder Society-Unified Parkinson's Disease Rating Scale; fMRI: Functional magnetic resonance imaging.

Five consecutive sessions (1 session per day) were performed on nonsimultaneous bilateral M1 (Fig. 1). A 70 mm air-cooled figure-eight coil (AirFilm® Coil, Magstim, Whitland, United Kingdom) connected to a Magstim Rapid2 Magnetic Stimulator (Magstim, Whitland, United Kingdom) was used for stimulation. We applied an iTBS protocol at 80% of the active motor threshold, which was obtained before the first iTBS session.

Both real iTBS and sham iTBS interventions adhered to the same procedure, although placebo stimulation was performed with an AirFilm SHAM coil (Magstim, Whitland, United Kingdom). Both coils were identical in terms of sound and sensation, as the sham coil generated clicking sounds and a mild sensory experience while producing no electric field.

Monophasic pulses were administered with the coil handle rotated 45° from the sagittal plane, eliciting anterior-posterior current flow in the first dorsal interosseous hotspot [29]. To ensure precise coil placement across all sessions, a 3D T1-weighted anatomical reconstruction image was used along with a frameless stereotaxic neuronavigation system (Brainsight, Rogue Research).

### Clinical-motor assessment: the MDS-UPDRS (parts II, III and IV)

The clinical assessment involved the administration of the MDS-UPDRS (parts II, III and IV) and Hoehn and Yahr scale. For the main clinical domains exploration – rigidity, bradykinesia, tremor and axial symptomatology – MDS-UPDRS part III subitems were clustered as follows: Rigidity, from item 3.3a to 3.3e; bradykinesia, from item 3.4a to 3.8b, summed to 3.14; tremor, from item 3.15a to 3.18; and axial symptoms, from item 3.10 to 3.13. Patients were assessed before and after iTBS treatment. Notably, the motor evaluation with MDS-UPDRS was longitudinally administered at four follow-up (post-1, post-2, post-3, and post-4) time points following the last iTBS session. These assessments were conducted immediately after the last iTBS intervention and again at one week, two weeks, and one month, providing information on the short-to mid-term evolution of motor outcomes following the treatment.

### Neurospsychiatric and cognitive assessment

All patients were assessed in different cognitive and neuropsychiatric domains using MMP [30], Frontal Assessment Battery [31], Benton Visual Retention Test (specifically, the M form) [32,33], Hamilton Depression Rating Scale (HDRS) [34], Hamilton Anxiety Rating Scale (HARS) [35], Starkstein Apathy Scale [36], Center for Neurologic Study-lability Scale [37]; Parkinson Fatigue Scale [38], Parkinson's Disease Questionnaire [39], Frontal Systems Behaviour Scale [40], Scale for the Evaluation of Neuropsychiatric Disorders in Parkinson's Disease [41] and Parkinson's Psychosis Questionnaire [42]. Neuropsychiatric and cognitive assessment was performed two weeks after the last iTBS intervention session.

### Sample stratification based on treatment response: responder/nonresponder categorization

Data were stratified into responders/nonresponders on the basis of clinical-motor, depressive and anxious symptomatology enhancement following real iTBS. Clinical motor responsiveness to iTBS was assessed using the total score of MDS-UPDRS Part III. A patient was classified as a *clinical-motor responder* if the average score across the four follow-up evaluations showed a reduction of >20% compared with baseline values following real iTBS while in the ON medication state [[43], [44], [45]]. A similar procedure, which was determined according to the minimum clinically important difference (MCID) criteria, was followed for the classification of depression and anxiety *responders/nonresponders*. The MCID is the smallest benefit a patient can perceive as meaningful after therapy or intervention. The optimal cut-off for detecting minimal clinically important improvement was 3 points for HDRS (depression) [46], [47] points for HARS (anxiety) [48] from baseline score. Importantly, analyses were restricted to responders to the real iTBS condition, given the limited number of participants showing motor or mood-related responses following sham stimulation.

### Resting-state fMRI: data acquisition

rs-fMRI scans were conducted using a T2-weighted functional echo-planar imaging sequence optimized for sensitivity to blood oxygen level-dependent signals. During the scans, participants remained with their eyes opened. The imaging parameters included a repetition time/echo time of 3000 ms/50 ms, 49 ascending slices, a matrix of 64 × 64 × 49, and a voxel size of 4.06 × 4.06 × 4.05 mm. Each session involved the acquisition of 120 vol over approximately 7 min.

Further information on image preprocessing is available in Supplementary material. rs-fMRI session was scheduled in the eighth day following the last iTBS session.

### Statistical analysis

Statistical analyses were performed using IBM SPSS Package version 23. Normality assumption data check of outcome variables was performed by employing the Shapiro‒Wilk test.

Post-hoc power estimation for the primary outcome (MDS-UPDRS III) was performed to contextualize detectability with the final sample size. Specifically, using the observed effect size (Cohen's d = 0.59; partial η^2^ = 0.27) and within-subject variance derived from the two-by-two crossover model, we estimated statistical power at approximately 60% (α = 0.05, two-tailed). This analysis is reported descriptively and is not intended to replace prospective sample size determination; This post-hoc estimate is reported descriptively only and should be interpreted with caution given the modest final sample size.

To account for potential carryover effects, differences in the baseline values of the variables of interest between Phase I and Phase II were analyzed. Additionally, possible order effects were examined through repeated-measures ANOVA with *treatment* (real iTBS, sham iTBS) and *time point* (baseline, post) as within-subject factors and *treatment order* (assigned treatment in phase I, real iTBS or sham iTBS) as a between-subjects factor.

To examine the effect of five bilateral M1-iTBS sessions on the UPDRS-MDS (part II, part III, part IV) and cognitive and neuropsychiatric data, we subsequently performed a series of two-way repeated measures ANOVAs. First, to study clinical-motor variables, *time point* (baseline, post-1, post-2, post-3, post-4) and *treatment* (real iTBS, sham iTBS) were included as within-subject factors. For the neuropsychiatric variables, *time point* (baseline, post) and *treatment* (real iTBS, sham iTBS) were used as within-subject factors. Mauchly's test was used to assess the assumption of sphericity. In the case of sphericity violation, Greenhouse-Geisser correction was used as appropriate. Statistical significance was set at p ≤ 0.05. Bonferroni's adjustment to alpha was applied for multiple comparisons to avoid Type I Error assumptions. To control type I error, Bonferroni correction was applied to (i) repeated-measures ANOVA analyses involving multiple subscales or clinical subdomains within the same instrument (i.e., MDS-UPDRS: parts II-IV; MDS-UPDRS part III clinical subdomains: bradykinesia, tremor, rigidity and axial symptoms; multidomain neuropsychiatric scales, such as PDQ-39 or PPQ); and (ii) rs-fMRI correlation analyses between clinical measures and FC Z-scores, with significance thresholds adjusted for the number of ROIs tested.

Student's paired *t*-test and independent-samples *t*-test were used for post-hoc analyses, as appropriate. Similar analyses were also performed for responders and nonresponders subgroups to real iTBS.

### Sample stratification based on treatment response: responder/nonresponder categorization

For resting-state FC (rs-FC) group-level analyses, FC maps were processed using tools such as SPSS and Statistical Parametric Mapping version 12 (SPM12) implemented in MATLAB 2022b (The Mathworks Inc., Sherborn, MA, USA). Voxelwise FC maps were created using AAL3 atlas parcellation, which was based on bilateral cortico-subcortical symptom-related PD ROIs. Seed regions were chosen to represent nodes within the motor circuit, mesocorticolimbic circuit and a set of intermediate regions functionally implicated in these networks. In the context of PD, intermediate regions were defined as those brain regions involved in the pathophysiology of both motor and affective symptoms. As implemented in SPM12, a flexible factorial design was used for a voxelwise brain analysis, where *treatment* condition (real iTBS, sham iTBS) and *time point* (baseline, post) were included as within-subject factors. Contrasts were set to evidence the interaction effect between factors (*treatment∗time point*). The significance threshold was set at a voxel-level uncorrected value of p ≤ 0.001 and a cluster-level whole-brain false discovery rate corrected (FDRc) value of p ≤ 0.05 with a minimum cluster size of k ≥ 15. This statistical analysis was complemented with a subsidiary t-paired test for post hoc exploratory analysis. A small volume correction was applied using a mask derived from flexible factorial significant contrast. No additional correction across ROIs was applied, given the hypothesis-driven ROI framework and the exploratory nature of these analyses; therefore, the findings should be interpreted as hypothesis-generating.

In addition, to further explore real iTBS treatment-induced changes from baseline to posttreatment, paired t-tests were employed for statistical analysis in subgroups of responders/nonresponders on the basis of clinical-motor, depressive, and anxious symptomatology enhancement. Results were thresholded at a voxel-level uncorrected value of p ≤ 0.001 and a cluster-level whole-brain false discovery rate corrected (FDRc) value of p ≤ 0.05 with a minimum cluster size of k ≥ 15. This analysis was not repeated for sham iTBS responder subgroups because the sample size was insufficient for valid statistical analysis in the responder subgroup. Age, disease duration, levodopa daily dosage, and MMP score were included as covariates in all analyses.

### Associations between clinical outcomes and resting-state functional connectivity

To evaluate whether clinical-motor and neuropsychiatric improvements in iTBS real responders (clinical-motor, depression and anxiety real iTBS responders’ subgroups, respectively) correlated with alterations in rs-FC, bivariate correlations were performed on covariate-adjusted residuals. Adjusted Z-scores values for rs-FC were obtained from eigenvariate function in SPM12. To minimize type I error, only FC maps showing significant Z-Score were included in the correlation analyses. In the case of clinical-motor responders to real iTBS, the FC maps were correlated with the clinical domain accounting for the largest proportion of the total percentage change, defined as [(average baseline domain score – average post iTBS domain score)/(average total baseline score) – (average total post-iTBS score)] ∗ 100. Age, MMP, disease duration and LEDD were included as covariates. Statistical significance was set at p ≤ 0.05, and Bonferroni alpha adjustment for multiple comparisons was employed.

## Results

No significant carryover effects were identified across any outcome measures (clinical, neurophysiological, or neuroimaging parameters), and the 3-month washout interval proved adequate to eliminate potential order-of-treatment effects, with no significant differences in baseline values or treatment responsiveness between the two study phases.

### Clinical-motor assessment: the MDS-UPDRS (parts II, III and IV)

#### Clinical-motor assessment: full sample analyses

In the full sample, two-way repeated-measures ANOVA (Supplementary Table 1) revealed an interaction effect (*treatment∗time point*) for MDS-UPDRS part III (F_1,14_ = 5.235, p = 0.014), along with a main *time point* main effect (F_1,14_ = 14.883, p ≤ 0.001). A main effect of *time point* was also observed for part IV (F_1,14_ = 6.532, p = 0.010), whereas no significant effects emerged for part II.

Exploratory within-group post-hoc analyses for MDS-UPDRS parts II and IV revealed some significant improvements following real iTBS and sham iTBS, respectively. Remarkably, this effect corresponded to an improvement of approximately 1 point, which did not reach significance when post-hoc between group comparisons were performed (Table 2).

**Table 2:** MDS-UPDRS follow-up evaluations for iTBS sham and iTBS real.

	MDS-UPDRS part II			MDS-UPDRS part III			MDS-UPDRS part IV		
(A) MDS-UPDRS part II, III and IV follow-up evaluations for iTBS sham and iTBS real									
	Sham iTBS	Real iTBS	ΔSham vs ΔReal p-value	Sham iTBS	Real iTBS	ΔSham vs ΔReal p-value	Sham iTBS	Real iTBS	ΔSham vs ΔReal p-value
**Baseline**	9.60 ± 5.96	10.53 ± 5.18		24.93 ± 6.96	28.6 ± 11.13		5.73 ± 3.26	5.47 ± 3.29	
**Post-1**	9.27 ± 6.08**^b^**	10.07 ± 5.55	0.728	22.33 ± 8.11**^a^**	21.60 ± 11.62**^a^**	**0.037∗**	4.73 ± 2.82**^a^**	4.93 ± 2.92	0.372
**Post-2**	9.47 ± 6.33	9.73 ± 5.67**^b^**	0.199	23.53 ± 9.78	20.47 ± 10.84**^a^**	**0.017∗**	4.73 ± 2.60**^a^**	4.80 ± 2.98	0.534
**Post-3**	9.33 ± 6.37	9.60 ± 5.70**^a^**	0.245	21.87 ± 7.74**^a^**	19.93 ± 10.07**^a^**	**0.033∗**	4.60 ± 2.56**^a^**	4.87 ± 3.01	0.407
**Post-4**	9.67 ± 6.44	9.60 ± 5.72**^a^**	0.173	22.87 ± 7.74**^b^**	20.80 ± 10.07**^a^**	**0.006∗**	4.40 ± 2.38**^a^**	4.73 ± 2.94	0.334
**Mean post**	9.43 ± 6.28	9.75 ± 5.61**^a^**	0.198	21.95 ± 7.92**^a^**	21.40 ± 10.70**^a^**	**0.036∗**	4.62 ± 2.56**^a^**	4.83 ± 2.96	0.397


(B) MDS-UPDRS part II, III and IV follow-up evaluations for iTBS real responders vs nonresponders									
	Nonresponders	Responders	ΔNonresponders vs ΔResponders p-value	Nonresponders	Responders	ΔNonresponders vs ΔResponders p-value	Nonresponders	Responders	ΔNonresponders vs ΔResponders p-value
**Baseline**	9.75 ± 4.50	10.82 ± 5.58		33.75 ± 14.73	26.73 ± 9.70		4.50 ± 1.73	5.82 ± 3.71	
**Post-1**	9.75 ± 4.50	10.18 ± 6.08	0.489	33.25 ± 15.69	17.36 ± 6.41**^a^**	**0.006∗**	4.50 ± 1.73	5.09 ± 3.30	0.661
**Post-2**	9.25 ± 5.85	9.91 ± 5.89**^a^**	0.851	30.75 ± 11.59	16.73 ± 8.16**^a^**	0.056	4.50 ± 1.73	4.91 ± 3.39**^b^**	0.343
**Post-3**	9.00 ± 6.06	9.82 ± 5.86**^a^**	0.949	31.25 ± 10.78	15.82 ± 6.10**^a^**	**0.006∗**	4.50 ± 1.73	5.00 ± 3.49	0.489
**Post-4**	9.50 ± 5.07	9.64 ± 6.17**^b^**	0.343	31.00 ± 10.23	17.09 ± 7.33**^a^**	**0.026∗**	4.50 ± 1.91**^a^**	4.82 ± 3.31**^a^**	0.280
**Mean post**	9.38 ± 5.33	9.89 ± 9.96**^a^**	0.661	31.56 ± 11.95	17.70 ± 7.82**^a^**	**0.040∗**	4.50 ± 1.74**^b^**	4.95 ± 3.36**^b^**	0.343

**Table A:MDS-UPDRS part II, part III and part IV scores at different follow-up time points for sham and real iTBS**. Full sample, n = 15; **Table B:MDS-UPDRS part II, part III and part IV scores at the different follow-up timepoints for clinical-motor responders and nonresponders to real iTBS**. Clinical-motor responders to real iTBS, n = 11; clinical-motor nonresponders, n = 4. Values are shown as mean ± sd. Statistical significance was set at p ≤ 0.05. **(∗)** indicates statistical significance in Δ (delta) analysis; (**a**) indicates statistical significance to baseline values; (**b**) indicates a trend towards significance to baseline values. For 2A, Δ values represent [Sham (baseline - post)] vs [Real (baseline - post)]. For 2B, Δ values represent [nonresponders (baseline - post)] vs [responders (baseline - post)] following real iTBS. **Abbreviations:** MDS-UPDRS: The Movement Disorder Society-Unified Parkinson's Disease Rating Scale. **Note:** Post-iTBS evaluation time points (post 1–4) were defined as assessments conducted immediately, at one week, two weeks and one month after iTBS.

**Fig. 2:**
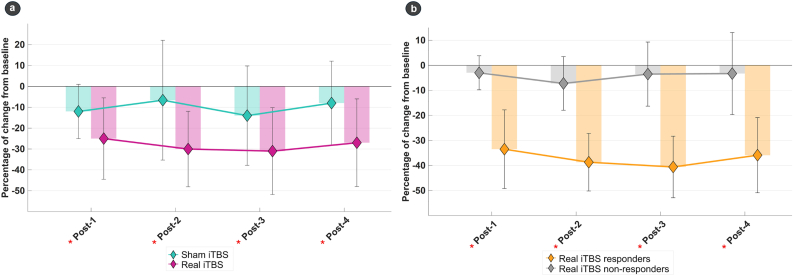
**Percentage change from baseline in MDS-UPDRS part III scores across the different follow-up evaluations**. Fig. 2**-a**: Analysis was performed for between-group [Sham (baseline - post)] vs [Real (baseline - post)]. Full sample, n = 15. Fig. 2**-b**: Analysis was performed for between-group [nonresponders (baseline - post)] vs [responders (baseline - post)]. Clinical–motor responders, n = 11; clinical-motor nonresponders, n = 4. Data are expressed as mean ± sd. Statistical significance was set at p ≤ 0.05 and indicated with a red asterisk (∗). For **a** and **b**, percentage change was calculated as [[(baseline score – post iTBS score) ∗ 100] −100]. **Abbreviations:** MDS-UPDRS: The Movement Disorder Society-Unified Parkinson's Disease Rating Scale. **Note:** Post-iTBS evaluation time points (post 1–4) were defined as assessments conducted immediately, at one week, two weeks and one month after iTBS.

Post hoc paired t-tests for MDS-UPDRS part III scores revealed that both treatment conditions led to significant mean changes from baseline (real iTBS, p = 0.001; sham iTBS, p = 0.016). Real iTBS resulted in significant improvement individually at each follow-up time point, whereas sham iTBS resulted in significant changes only at the immediate evaluation and at the post-3 follow-up time point (Table 2). The greatest improvement was observed at post-3 in both conditions, real iTBS (8.90 ± 6.11; 30.7%) and sham iTBS (2.87 ± 5.33; 14.3%) (Fig. 2). The magnitude of average improvement was significantly greater following real iTBS, with a mean difference of 5 points compared to sham (p = 0.036).

Subsequently, a clinical subdomain-level analysis of the MDS-UPDRS part III was performed. Two-way repeated-measures ANOVA revealed significant main *time point* effect for bradykinesia (F_4,14_ = 5.836, p = 0.001), tremor (F_4,14_ = 3.842, p = 0.008), axial (F_4,14_ = 4.261, p = 0.004) and rigidity (F_4,14_ = 9.494, p ≤ 0.001) symptomatology. In addition, significant interaction effect (*treatment∗time point*) was found for bradykinesia (F_4,14_ = 3.809; p = 0.008) and tremor (F_4,14_ = 2.884, p = 0.031). Accounting for the number of clinical subdomains composing the MDS-UPDRS part III scale (α/4 = 0.0125), these effects remained significant after Bonferroni correction except for *treatment∗time point* interaction in tremor clinical subdomain.

Post-hoc paired *t*-test analysis was exclusively conducted for bradykinesia domain, as it was the only *treatment∗time point* interaction effect that remained significant following Bonferroni correction, revealing a maximal between-treatment condition difference at the post-3, favouring real iTBS (p = 0.048). Further exploratory analyses for tremor, rigidity and axial symptomatology are also reported in Table 3.

**Table 3:** MDS-UPDRS part III follow-up evaluations for iTBS sham and iTBS real.

	BRADYKINESIA			TREMOR			RIGIDITY			AXIAL		
(A) MDS-UPDRS part III follow-up evaluations for iTBS sham and iTBS real												
	Sham iTBS	Real iTBS	ΔSham vs ΔReal p-value	Sham iTBS	Real iTBS	ΔSham vs ΔReal p-value	Sham iTBS	Real iTBS	ΔSham vs ΔReal p-value	Sham iTBS	Real iTBS	ΔSham vs ΔReal p-value
**Baseline**	11.47 ± 4.94	13.60 ± 6.59		2.47 ± 2.36	3.87 ± 3.27		5.87 ± 3.13	5.73 ± 3.77		3.13 ± 1.99	3.47 ± 2.42	
**Post-1**	10.33 ± 5.65	10.33 ± 7.03	**0.049∗**	1.73 ± 2.19	2.00 ± 2.00	0.143	5.47 ± 3.10	5.13 ± 3.70	0.638	2.80 ± 2.04	2.53 ± 1.85	0.189
**Post-2**	11.33 ± 6.07	9.93 ± 5.91	**0.012∗**	2.60 ± 2.38	2.07 ± 2.28	0.111	5.27 ± 3.09	5.00 ± 3.84	0.843	2.47 ± 1.96	2.00 ± 1.81	0.111
**Post-3**	10.87 ± 6.25	9.80 ± 5.94**^a^**	**0.048∗**	2.53 ± 2.26	2.00 ± 2.04**^a^**	0.066	4.73 ± 2.63**^b^**	4.53 ± 3.80**^a^**	0.890	2.27 ± 1.62**^a^**	2.20 ± 1.66**^a^**	0.361
**Post-4**	11.20 ± 5.31	10.20 ± 5.34	**0.011∗**	2.73 ± 2.19	1.67 ± 1.99	**0.006∗**	4.93 ± 3.17	4.87 ± 3.68	0.898	2.00 ± 1.25	2.47 ± 1.77	0.764
**Mean post**	10.93 ± 5.61	10.07 ± 5.87**^a^**	**0.014∗**	2.40 ± 1.81	1.93 ± 1.86**^a^**	**0.035∗**	5.10 ± 2.83**^b^**	4.89 ± 3.70**^a^**	0.852	2.38 ± 1.63**^a^**	2.30 ± 1.66**^a^**	0.295

**(B) MDS-UPDRS part III follow-up evaluations for real responders vs nonresponders**												

	**Nonresponders**	**Responders**	**ΔNonresponders vs ΔResponders p-value**	**Nonresponders**	**Responders**	**ΔNonresponders vs ΔResponders p-value**	**Nonresponders**	**Responders**	**ΔNonresponders vs ΔResponders p-value**	**Nonresponders**	**Responders**	**ΔNonresponders vs ΔResponders p-value**

**Baseline**	17.50 ± 7.94	12.18 ± 5.79		1.75 ± 1.71	4.64 ± 3.41		9.00 ± 4.25	4.55 ± 2.94		3.50 ± 3.00	3.45 ± 2.34	
**Post-1**	17.00 ± 8.91	7.91 ± 4.59	**0.039∗**	1.50 ± 1.92	2.18 ± 2.09	**0.013∗**	9.25 ± 4.11	3.63 ± 2.20	0.165	3.50 ± 3.00	2.18 ± 1.25	**0.018∗**
**Post-2**	15.75 ± 6.24	7.82 ± 4.33	0.136	1.75 ± 3.50	2.18 ± 1.89	0.070	8.50 ± 3.87	3.73 ± 3.07	0.744	2.75 ± 2.22	1.73 ± 1.68	0.210
**Post-3**	16.25 ± 6.08	7.46 ± 3.96**^a^**	0.140	1.50 ± 2.38	2.18 ± 1.99**^a^**	0.075	8.50 ± 3.87	3.09 ± 2.66**^a^**	0.283	3.00 ± 2.00	1.91 ± 1.51**^a^**	0.170
**Post-4**	15.75 ± 5.56	8.18 ± 3.71	0.347	1.00 ± 1.41	2.09 ± 2.34	0.159	9.00 ± 2.95	3.36 ± 2.66	0.203	3.25 ± 2.50	2.18 ± 1.47	0.261
**Mean post**	16.19 ± 6.65	7.94 ± 3.81**^a^**	0.103	1.44 ± 2.26	2.11 ± 1.78**^a^**	**0.013∗**	8.81 ± 3.67	3.46 ± 2.60**^a^**	0.275	3.13 ± 2.42	2.00 ± 1.32**^a^**	0.138

**Table A**: **MDS-UPDRS part III domain (bradykinesia, tremor, rigidity and axial symptoms) scores at the different follow-up time points for iTBS sham and iTBS real groups.** Full sample, n = 15. **Table B:MDS-UPDRS part III domain (bradykinesia, tremor, rigidity and axial symptoms) scores at different follow-up time points for clinical-motor responders and nonresponders to real iTBS.** Clinical-motor responders to real-iTBS, n = 11; clinical-motor nonresponders, n = 4. Values are shown as mean ± sd. Statistical significance was set at p ≤ 0.05. (∗) indicates statistical significance in Δ (delta) analysis; (**a**) indicates statistical significance to baseline values; (**b**) indicates a trend towards significance to baseline values. For 2A, Δ values represent [Sham (baseline - post)] vs [Real (baseline - post)]. For 2B, Δ values represent [nonresponders (baseline - post)] vs [responders (baseline - post)] following real iTBS. **Abbreviations:** MDS-UPDRS: The Movement Disorder Society-Unified Parkinson's Disease Rating Scale. **Note:** Post-iTBS evaluation time points (post 1–4) were defined as assessments conducted immediately, at one week, two weeks and one month after iTBS.

### Clinical-motor responsiveness to real iTBS

Clinical-motor responsiveness analyses were subsequently conducted for the real iTBS condition. Eleven patients were subsequently classified as real iTBS clinical-motor responders. No significant between-group differences emerged between real iTBS clinical-motor responders and nonresponders in the MDS-UPDRS part II or part IV (Table 2). In contrast, for the MDS-UPDRS part III, real iTBS clinical-motor responders showed a significantly greater mean improvement from baseline (17.70 ± 7.82; 37.18%) than nonresponders (31.56 ± 11.95; 4.25%, p = 0.001) (Fig. 2). This improvement was consistently significant across all four MDS-UPDRS part III assessments in the real iTBS clinical-motor responder group, whereas clinical-motor nonresponders did not show significant changes over time. Between-group comparisons further revealed greater motor improvement in real iTBS clinical-motor responders across all time points, except for post-2 (α = 0.056), where only a trend towards significance was observed (Table 2).

Finally, analyses of the MDS-UPDRS part III subdomains for real iTBS clinical-motor responders were conducted. Exploratory analyses at the time point of maximal clinical benefit (post-3) indicated that, among real iTBS clinical-motor responders, bradykinesia (35.29%) emerged as the most amenable to improvement following real iTBS (axial: 17.34%; rigidity: 17.45%; tremor: 24.66%). Notably, all the clinical motor domains significantly changed from baseline, although no statistically significant differences were observed between responders and nonresponders (Table 3).

### Neuropsychiatric and cognitive assessment

#### Neuropsychiatric and cognitive assessment: full sample analyses

In the full sample, two-way repeated-measures ANOVA (Supplementary Table 2) was performed as appropriate. Significant *time point* main effects for HDRS (F_1,14_ = 10.499, p = 0.006), HARS (F_1,14_ = 13.180, p = 0.003) and PPQ sleep disturbances subscale (PPQ-sleep) (F_1,14_ = 5.527, p = 0.034) were observed. However, the PPQ-sleep effect did not remain significant after Bonferroni correction considering the number of PPQ subdomains analyzed (α/4 = 0.0125). Post-hoc analysis revealed a significant improvement after iTBS that was unrelated to treatment condition. Further analyses are shown in Supplementary Table 3.

**Fig. 3:**
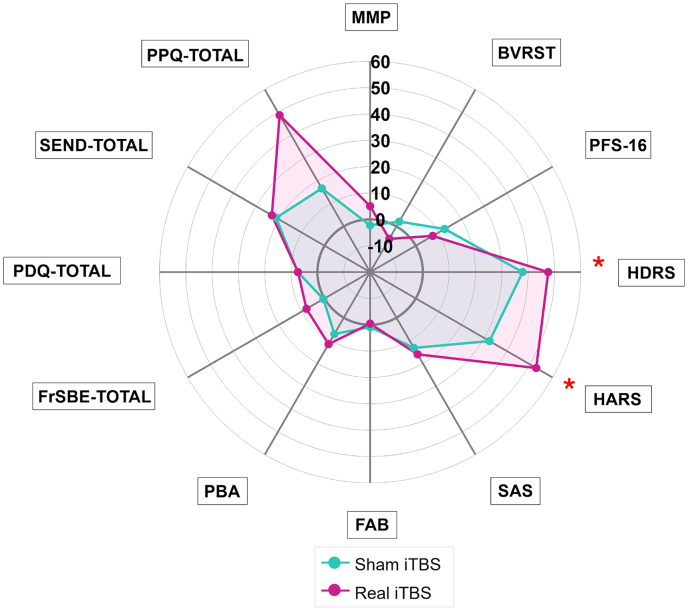
**Spiderplot: Direct score relative percentage change from baseline on MDS-UPDRS part III during the different follow-up evaluations following sham and real iTBS**. Full sample, n = 15. Direct score relative percentage of change was performed as [[(baseline score – post iTBS score) ∗ 100] −100]. Statistical significance was set at p ≤ 0.05 and indicated with a red asterisk (∗). Within-group analysis was performed (real iTBS, baseline vs. post; sham iTBS, baseline vs. post). **Abbreviations:** BVRB: Benton Visual Rating Battery; FAB: Frontal Assessment Battery; FrSBE-TOTAL: Frontal Systems Behavior Scale; HARS: Hamilton Anxiety Rating Scale; HDRS: Hamilton Depression Rating Scale; MMP: Mini Mental Parkinson; PFS-16: Parkinson's Fatigue Scale; PDQ-TOTAL: Parkinson's Disease Questionnaire Total score; PPQ-TOTAL: Parkinson's Psychosis Questionnaire – Total Score; SAS: Starkstein Apathy Scale; SEND-TOTAL: Scale for the evaluation of neuropsychiatric disorders in Parkinson's disease Total Score.

For clinical purposes, further post-hoc analyses were performed on variables that were statistically significant in the aforementioned factorial analysis. HDRS and HARS improved after iTBS, independently of the treatment conditions (real, sham iTBS). However, the change from baseline was statistically significant only for the real iTBS condition (HDRS: Relative change from baseline: 47.62%, t-value = 3.696, p = 0.002; HARS: Relative change from baseline: 37.93%, t-value = 3.172, p = 0.007), whereas only a statistically significant trend was obtained after sham iTBS (Fig. 3).

#### Mood-related responsiveness to real iTBS: depression and anxiety

Based on MCID criteria, we also determined real responders/nonresponders subgroups for both depressive (HDRS) and anxious (HARS) symptomatology**.** In line with this finding, real iTBS depression responders showed a mean score reduction of 4.9 (p = 0.016) points in direct score compared with only 0.8 (p = 0.034) points for nonresponders. In the same vein, real iTBS anxiety responders reached an improvement of 7.8 (p = 0.027) points in direct score, whereas nonresponders showed a modest nonsignificant 1-point (p = 0.088) change from baseline. Statistical significance between real responders and nonresponders was found for both Δ HDRS and Δ HARS scores (Δ = baseline direct score – post iTBS direct score; p ≤ 0.001).

### Voxelwise resting-state fMRI analyses

**Fig. 4:**
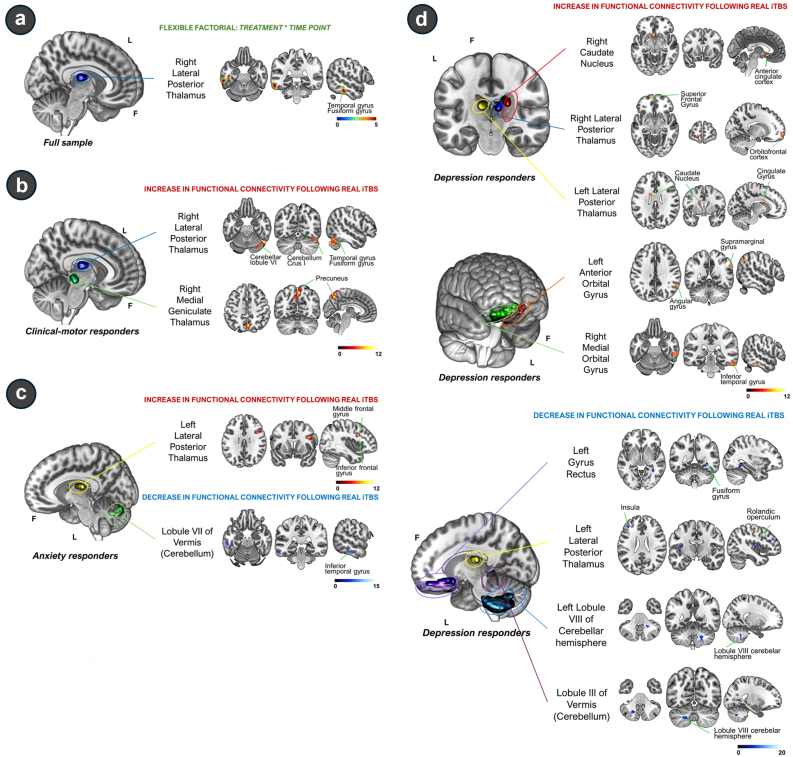
**Voxelwise results of resting-state fMRI.(a)Flexible factorial analysis**. **(b**–**d)Paired*****t*****-test results**. **(a)Full sample:** Sample size, n = 15. *Seed*: *Right lateral posterior thalamus* – cluster 1 (k = 147; [-60, −36, −18]; Z = 4.40): Left inferior temporal gyrus, middle temporal gyrus and fusiform gyrus. **(b) Real iTBS clinical-motor responders**: Sample size, n = 11. *Seed*: *Right lateral posterior thalamus* – cluster 1 (k = 88; [51, -72, -33]; Z = 4.82): Right cerebellum crus 1, right lobule VI of cerebellar hemisphere, fusiform gyrus, temporal inferior gyrus; *Seed*: *Right Medial Geniculate thalamus* – cluster 1 (k = 137; [6, -57, 72]; Z = 4.10): Right and left precuneus. **(c) Real iTBS anxiety responders**: Sample size, n = 6. *Seed*: *Left lateral posterior thalamus* – cluster 1 (k = 17; [39, 12, 27]; Z = 4.14): Left inferior frontal gyrus (opercular part) and middle frontal gyrus; *Seed*: *Lobule VII of vermis* – cluster 1: (k = 21; [-60, −36, −21]; Z = 3.71): inferior temporal gyrus. **(d) Real iTBS depression responders:** Sample size, n = 7. *Seed*: *Right caudate nucleus* – cluster 1 (k = 15; [-3, 15, −9]; Z = 3.87): Anterior cingulate; *Seed*: *Right lateral posterior thalamus* – cluster 1 (k = 15; [-15, 63, −9]; Z = 3.84): Left superior frontal gyrus and orbitofrontal cortex; *Seed*: *Left lateral posterior thalamus* – cluster 1: (k = 15; [-12, 0, 27]; Z = 3.91): Left caudate nucleus and left cingulate gyrus; *Seed*: *Left anterior orbital gyrus* – cluster 1 (k = 15; [54, -54, 39]; Z = 4.01): Right supramarginal gyrus and right angular gyrus; *Seed*: *Right medial orbital gyrus* – cluster 1 (k = 24; [54, -33, -24]; Z = 4.10): Inferior temporal gyrus; *Seed*: *Left gyrus rectus* – cluster 1 (k = 19; [33, -48, -6]; Z = 3.81): right fusiform gyrus; *Seed: Left lateral posterior thalamus* – cluster 1 (k = 15; [-42, −12, 12]; Z = 3.94): Left insula and left rolandic operculum; cluster 2 (k = 16; [-42, 39, 24]; Z = 4.16): Left inferior frontal gyrus (triangular part) and left middle frontal gyrus; *Seed*: *Left lobule VIII of cerebellar hemisphere* – cluster 1 (k = 15; [21, -51, -51]; Z = 4.63): Right lobule VIII of cerebellar hemisphere; *Seed*: *Lobule III of vermis* – cluster 1 (k = 15; [-21, −63, −42]; Z = 3.58): Left lobule VIII of cerebellar hemisphere. **Abbreviations:** F: Frontal; L: Left: iTBS: Intermittent theta burst stimulation. **Note:** (i) The color bar located at the lower right margin of each subpanel indicates the range of T-values; (ii) for each cluster, the corresponding anatomical regions are shown in at least one anatomical view (coronal, axial or sagittal); (iii) for A and B, the significance threshold was set at a voxel-level uncorrected value of p ≤ 0.001 and a cluster-level whole-brain false discovery rate corrected (FDRc) value of p ≤ 0.05 with a minimum cluster size of k ≥ 15.

In the full sample, flexible factorial results revealed a significant interaction effect between conditions (*treatment*∗*time point*) and driving effects from the right lateral posterior thalamus to the left inferior temporal gyrus (ITG), left middle temporal gyrus, and left fusiform gyrus (t = 4.67, pFDRc = 0.038) (Fig. 4). Post-hoc analysis with paired *t*-test is shown in Supplementary material.

#### Resting-state functional connectivity modulation in clinical-motor responders

Significant results of a voxelwise paired *t*-test in a subset of patients with PD (n = 11; Fig. 4), corresponding to real iTBS clinical-motor responders, revealed FC increase (paired *t*-test contrast, post real iTBS FC > baseline iTBS FC) between i) the right lateral posterior thalamus and a cluster of voxels in the right cerebellum, fusiform gyrus, and ITG (t = 11.15, pFDRc = 0.038), and ii) the right medial geniculate thalamus with the contralateral and ipsilateral precuneus (t = 7.40, pFDRc = 0.017).

#### Resting-state functional connectivity modulation in depression responders

Significant voxelwise paired *t*-test for real iTBS depression responders (n = 7) revealed a total of nine seeds with FC changes following real iTBS. The anatomical distribution of all significant FC changes is graphically represented in Fig. 4.

On the one hand, increased functional coupling (paired *t*-test contrast, post real iTBS FC > baseline iTBS FC) was observed between i) the right caudate nucleus and a cluster of voxels located in the anterior cingulate (t = 10.95, pFDRc ≤0.001); ii) the right lateral posterior thalamus and the left superior frontal gyrus and orbitofrontal cortex (OFC) (t = 10.75, pFDRc ≤0.001, k = 15); iii) the contralateral lateral posterior thalamus was associated with strengthened FC with left caudate nucleus and cingulate gyrus (t = 11.35, pFDRc = 0.001); iv) the left anterior orbital gyrus seed was correlated with increased FC within a cluster located in the right supramarginal and angular gyrus (t = 12.41, pFDRc = 0.001); and v) the right medial orbital gyrus and the ITG (t = 13.37, pFDRc ≤0.001).

On the other hand, a decrease in FC (paired *t*-test contrast, baseline FC > post real iTBS FC) was also detected between different regions for real iTBS depression responders: i) the left gyrus rectus correlated with lower FC within a cluster in the right fusiform gyrus (t = 10.40, pFDRc ≤0.001); ii) the left lateral posterior thalamus manifested reduced FC with two clusters of voxels, the first located within the left insula (t = 11.68, pFDRc ≤0.001) and the second, located in the left frontal gyrus (t = 14.10, pFDRc ≤0.001); and iii) seeds located within the left lobule VIII of cerebellar hemisphere and lobule III of the vermis exhibited lower local FC within the intracerebellar regions (t = 21.92, pFDRc = 0.001; and t = 8.65, pFDRc = 0.001).

### Resting-state functional connectivity modulation in anxiety responders

Significant voxelwise paired *t*-test for real iTBS anxiety responders (n = 6) revealed a total of two seeds (Fig. 4) with FC changes following real iTBS.

Increased functional coupling (paired *t*-test contrast, post real iTBS FC > baseline iTBS FC) was observed between i) the left lateral posterior thalamus and the left inferior frontal gyrus (opercular part) and middle frontal gyrus (t = 20.38, p ∼ FDRc∼ ≤ 0.001). Additionally, a decrease in FC (paired *t*-test contrast, baseline FC > post real iTBS FC) was detected between i) the seed located in lobule VII of the vermis and the ITG (t = 12.86, p ∼ FDRc∼ ≤ 0.001). More detailed anatomical information for cluster location and graphical representation is provided in Fig. 4.

### Imaging correlates of clinical-motor and neuropsychiatric improvement

**Fig. 5:**
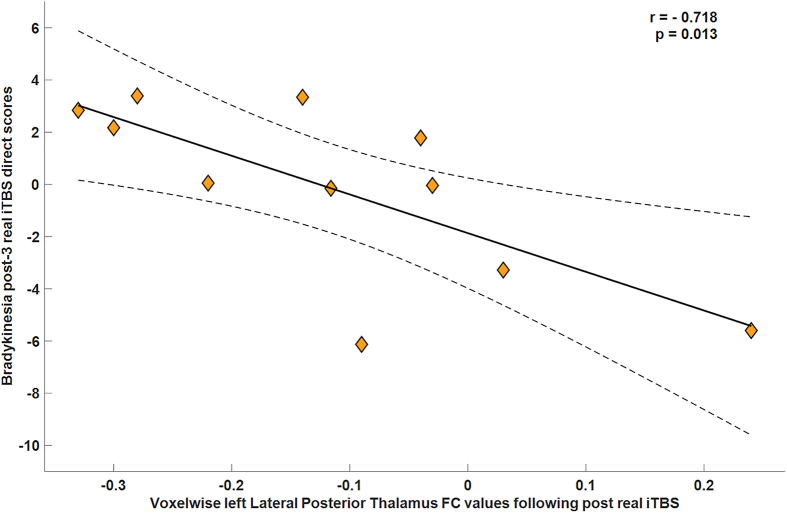
**Relationship between right medial geniculate thalamus and bradykinesia score following real iTBS.** Scatterplot representing the relationship between the right medial geniculate thalamus and bradykinesia score following real iTBS in clinical motor responders (n = 11). Bivariate Pearson correlation analyses for covariate-adjusted residuals were used. Age, disease duration, levodopa equivalent daily dose and Mini-Mental Parkinson's baseline score from phase I were used as covariates. Line indicates the linear regression, and the shaded area represents the 95% confidence interval. **Abbreviations:** iTBS: intermittent theta burst stimulation; FC: functional connectivity. **Note:** Post-3 evaluation was scheduled two-weeks following the last iTBS session.

Pearson correlation analyses were performed to explore the relationship between the right medial geniculate thalamus, right lateral posterior thalamus rs-FC and bradykinesia subitem scores before and after iTBS treatment in real iTBS motor responders. For the former, the posttreatment correlation was significant (r = −0.718; p = 0.013) and survived Bonferroni correction for multiple comparisons (α/2 = 0.025) (Fig. 5). In contrast, no significant relationship was observed at baseline (r = 0.240, p = 0.478). Similarly, no significant association was found between the right lateral posterior thalamus and bradykinesia score at either time point. Correlation analyses of depression and anxiety responders were not conducted because of the small sample size.

## Discussion

In this study, we have shown that M1-iTBS exerts a dual-action therapeutic effect in patients with mid-stage PD, simultaneously reducing both motor disability and depression/anxiety mood-related symptoms. Our one-month follow-up data revealed not only symptom-related enhancement but also cortico-subcortical functional reorganization, likely subserving motor improvement and mood regulation. These findings support the depiction of the brain as a large-scale interconnected network organized into functional circuits and reinforce the notion that TBS can modulate regional and network dynamics, even beyond the stimulated area. By integrating behavioural outcomes with objective large-scale neuroimaging evidence, this study elucidates how M1-iTBS exerts therapeutic effects in patients with PD through adaptative reorganization of interconnected circuits. precuneus.

### Motor symptomatology

Regarding the full sample, real iTBS intervention resulted in rapid motor improvement after the immediate posttreatment evaluation (post-1), with progressive gains through the different longitudinal follow-up time points and reaching maximal motor score benefit (9-point reduction) at the second week (post-3). For real iTBS clinical-motor responders, the magnitude of benefit reached 11 points [9,49], which clearly surpasses prior reports—where average improvements rarely exceed 6 points—and doubles the anchored 5-point MCID criteria for moderate improvements, underscoring the relevance of the observed clinical benefit in our sample [50]. Additionally, the absence of apparent improvement in MDS-UPDRS part II scores likely reflects the limited power for short-term assessments rather than a real impact on functional motor benefit. With respect to the MDS-UPDRS part IV, scores remained stable, suggesting that the ON state was optimal and unrelated to dyskinesia.

With respect to rs-FC in real iTBS clinical-motor responders, we observed that iTBS-induced modulation of FC emanated prominently from the posterior lateral thalamus, a key associative hub that integrates sensory and visuocognitive information with motor outputs [51]. Disruptions in thalamo-cortical circuitry, particularly involving posterior thalamic nuclei, are increasingly recognized as a core feature of PD. These alterations contribute not only to motor dysfunction but also to deficits in sensorimotor integration and cognitive processing [52,53].

In real iTBS clinical-motor responders, enhanced FC between cerebellum and posterior thalamic nodes suggests a reinforcement of compensatory cerebello-thalamo-cortical mechanisms. This process likely drives the observed clinical improvement and is consistent with prior evidence demonstrating the ability of iTBS to modulate cerebellar-thalamic and thalamo-cortical pathways [54].

The cerebellum plays a central role in PD pathophysiology [[55], [56], [57]], and cerebello-thalamic interactions are known to be disrupted secondary to BG dysfunction [[58], [59], [60], [61], [62]]. In this context, our findings suggest that the therapeutic effects of iTBS may be partially mediated by restoring the functional integrity of thalamo-cortical pathways. This effect may be particularly relevant for associative thalamic territories such as the lateral posterior nucleus, which are implicated in both compensatory and pathological mechanisms of PD symptomatology.

Beyond cerebellothalamic strengthening in clinical responders, enhanced FC also emerged between thalamic and visual-associative cortices. In particular, geniculate‒precuneal coupling was strengthened, suggesting a restoration of thalamo-cortical communication within sensory-integrative networks known to be disrupted in PD [[63], [64], [65]]. This strengthening may stem from enhanced proprioceptive processing [66], given the role of the precuneus in visuospatial processing. Such changes may facilitate higher-order cognitive functions, including action planning and motor imagery [67], ultimately contributing to enhanced postural and gait control [66].

Notably, selective precuneal inhibition has been shown to impair gait control, indicating that geniculate–precuneal coupling may facilitate more coordinated movement execution and postural stability [68]. In line with this, previous neuroimaging studies have shown that patients with PD may exhibit reduced activation of the superior parietal lobule, a region known to mediate kinesthetic sensation [69]. This region contributes to monitoring limb position during internally guided actions and to adjusting movement parameters in the absence of external cues [70,71]. These functions are impaired in bradykinesia, and its functional reorganization may have partially contributed to the underlying motor improvement observed in this clinical subdomain.

In clinical responders, the reduction in bradykinesia was associated with strengthened functional connectivity between the medial geniculate thalamus and the precuneus. These findings may reflect a functional reinforcement of thalamo-parietal coupling, which is involved in sensory integration and kinesthetic control during motor execution. This mechanism may partially compensate for dysfunctional motor thalamic processing.

Collectively, this functional reorganization provides evidence that iTBS modulates cerebello-thalamo and thalamo-cortical connectivity, enabling the integration of sensorimotor and associative enhanced processing [72] and thereby providing a mechanistic substrate for substantial motor improvement following iTBS [73,74].

### Depression and anxiety symptoms

Both iTBS treatment conditions improved depressive and anxiety symptoms; however, real iTBS demonstrated greater efficacy, with reductions of 3.7 and 6 points on the HDRS and HARS, respectively. Prior evidence has shown that targeting motor areas, such as M1, can alleviate depression and related symptoms [75,76]. Recently, a meta-analysis revealed that bilateral M1 stimulation has a beneficial effect on patients with PD depression and that combined rTMS-pharmacotherapy approaches are effective at reducing anxiety [77]. These observations suggest that M1 modulation extends beyond motor circuits and can therefore contribute to mood regulation.

In real iTBS depression responders, OFC-limbic downregulation, evidenced by reduced FC between gyrus rectus and fusiform gyrus, may reflect attenuation of maladaptive ruminative processing and self-referential bias [78,79]. Concurrently, decreased thalamic–insular coupling may reduce maladaptive salience and interoceptive signalling with negative affect [80]. This change may potentially contribute to reduced emotional hypervigilance and negative affective misattribution.

Conversely, enhanced striatal-thalamic-cingulate connectivity [[81], [82], [83], [84]] suggests a strengthening of reward-motivational circuits. This reconfiguration may foster goal-directed behaviours and cognitive preservation [85], a pattern robustly associated with depressive improvement [86], and that may underlie adaptive affective regulation.

Additionally, depression responders also exhibited increased thalamic coupling with frontal-dorsomedial regions. This pattern may promote emotional flexibility and adaptive regulation [87]. In parallel, local cerebellar FC reorganization reinforces the growing view of the cerebellum as an integral node within affective circuits, further contributing to depressive symptomatology alleviation [[88], [89], [90]].

Overall, depression responders exhibited patterns consistent with widespread functional reconfiguration within cortico-striato-thalamic loops. Our findings suggest the involvement of both top-down and bottom-up regulatory mechanisms converging into enhanced affective processing. Taken together, these bidirectional changes may reflect a shift toward more adaptive cortico-striato-thalamo-limbic interactions and provide a mechanistic basis for the observed mood improvements in depression responders.

Additionally, anxiety responders exhibited a dual regulatory mechanism. First, reduced cerebellar vermis VII-inferior temporal gyrus connectivity may reflect diminished maladaptive coupling associated with emotional hypervigilance and aberrant limbic processing [[91], [92], [93], [94]]. Second, enhanced fronto-thalamic coupling—specifically between lateral posterior thalamus and inferior (IFG) and middle frontal gyrus—suggests adaptive recruitment of prefrontal inhibitory control networks [95]. Previous studies have demonstrated that aberrant opercular connectivity within salience circuits seems to characterize anxious states [96,97], whereas dorsolateral prefrontal hypoactivity impairs top-down limbic regulation [86,98]. Moreover, increased IFG opercular connectivity likely reflects strengthened prefrontal inhibitory capacity, counteracting limbic overactivity. Dysregulated IFG dynamics have also previously been linked to weakened excitatory ventromedial prefrontal projections and aberrant inhibition of amygdala function [99].

Collectively, these findings indicate that anxiety improvement in patients with PD may arise from dual mechanisms. These include the reduction and potential normalization of maladaptive cerebellar-limbic dysregulation, along with strengthening of prefrontal top-down regulatory control over limbic circuits.

### General mechanisms of iTBS and functional connectivity

Bilateral M1-iTBS-induced motor and nonmotor improvements likely arise through multiple converging mechanisms. M1 presents both direct and indirect structural connectivity with motor and nonmotor cortical regions—the cerebellum, prefrontal, somatosensory, and cingulate cortex [89,100,101]—and subcortical structures, including the subthalamic nucleus and globus pallidus [102,103]. M1 connectivity to subcortical structures via white matter tracts allows stimulation effects to propagate across distributed networks, thereby driving modulation of FC patterns [17,104].

Additionally, rTMS/TBS modulates neural synchrony within cortico-subcortical circuits, particularly along the M1-subthalamic nucleus pathway, thereby attenuating maladaptive oscillatory activity [20,[105], [106], [107]]. Within this framework, M1 connectivity with the subthalamic nucleus via the hyperdirect pathway [19,20] may represent a strategic target for the mitigation of motor and nonmotor symptoms in patients with moderate-to-advanced PD.

Within this network-level propagation of iTBS effects, it is important to clarify that the direction of rs-FC change should not be interpreted uniformly as beneficial or detrimental. Rather, increased connectivity may reflect adaptive reintegration within underconnected circuits, whereas decreased connectivity may indicate attenuation of maladaptive hyperconnectivity or inefficient compensatory recruitment. Interpretation may depend on the functional role of the network involved.

Beyond network-level effects, several neural mechanisms support the efficacy of iTBS. Brain homeostasis may drive compensatory functional reorganization, as interconnected regions adaptively respond to stimulation-induced modulation [108]. Underlying these adaptive changes, iTBS facilitates long-term potentiation (LTP) induction [109,110] through glutamatergic and N-methyl-D-aspartate receptor activation, restoring the excitatory-inhibitory neural balance [111].

In addition, bilateral M1 iTBS may promote the release of brain-derived neurotrophic factor liberation, supporting both acute transmission and LTP-like synaptic strengthening [112]. Furthermore, the increase in striatal dopamine release induced by iTBS could restore corticostriatal transmission [113] and improve both motor and nonmotor function [10,11]. To this, we must add the involvement of other neurotransmitters in additional modulatory pathways of the motor cortex, such as the raphe nuclei (serotonin), the locus coeruleus (norepinephrine), and the nucleus basalis of Meynert (acetylcholine) [7]. which are essential for the control of nonmotor symptoms [77]. These functional mechanisms are further substantiated by complementary structural findings demonstrating that responders to bilateral M1-iTBS exhibit increased grey matter volume in the ventral diencephalon and elevated serum BDNF concentrations [114]. Together, these findings suggest that iTBS-induced clinical benefits are mediated by both circuit-level functional reorganization and structural neuroplasticity [113,114]. In summary, these neurochemical and network mechanisms converge synergistically to mediate the therapeutic effects of iTBS.

Despite its rigorous design and execution, this study has several limitations. Sample heterogeneity in clinical phenotype and disease duration should be considered when interpreting the data. Moreover, although consistent with prior iTBS studies in PD, the sample size remains modest. This may limit the stability, precision and generalizability of some of observed effects, particularly in the responder subgroup analyses and exploratory imaging findings. Although post hoc power analysis confirmed adequate sensitivity for the primary motor outcome, the results should be interpreted with caution. Smaller samples increase the risk of both type I and type II errors and reduce the generalizability of network-level inferences. Nonetheless, the double-blind crossover design and the inclusion of a broad range of clinical measures strengthen the robustness of our study. However, replication in larger cohorts may be recommendable.

In addition, patients with cognitive impairment were excluded from this study for methodological and safety reasons, despite representing a clinically meaningful subgroup within the PD population. Future studies with larger samples would enable stratified analyses to better characterize how disease progression and clinical subtype modulate responsiveness to iTBS.

Importantly, mood outcome improvements following both treatment conditions likely reflect multiple confounding factors. Patient-reported mood measures are particularly vulnerable to social desirability bias, especially in the context of novel interventions such as iTBS. Participants' expectations may therefore lead to an overestimation of self-reported symptom improvement, independently of genuine therapeutic benefit. This bias is especially relevant for mood assessment, where subjective perception substantially influences scale scores.

While rs-fMRI provides objective neuroimaging evidence, future studies should incorporate objective behavioural measures to validate subjective mood gains. Additionally, conducting OFF-medication assessments would help disentangle intrinsic TBS-induced effects from dopaminergic medication interactions, particularly for mood-related symptoms where dopamine-monoamine involvement is critical. Finally, the one-month follow-up in this study was relatively brief, capturing only short- and mid-term clinical changes, as well as short-term alterations in neuropsychiatric and resting-state functional connectivity. Consequently, the durability of these improvements and the long-term trajectory of network reorganization remain uncertain. Longer-term studies are needed to determine whether these effects persist, evolve, or diminish over time.

It is important to note that the interpretation of these findings is necessarily informed by the broader rTMS literature, as iTBS studies in PD remain scarce, particularly those addressing FC changes or mood-related outcomes following M1 stimulation. Consequently, some extrapolation from traditional rTMS evidence has been unavoidable, underscoring a critical gap in the field and reinforcing the need for dedicated iTBS trials in this population.

Overall, this study provides evidence that bilateral M1-iTBS exerts dual therapeutic benefit, namely marked motor symptom alleviation coupled with clinically modest mood improvement, through functional reorganization within interconnected cortico-striato-thalamic networks. These findings support M1-iTBS as a mechanistically grounded and effective adjunctive intervention for multimodal PD symptom management, warranting further integration in larger-scale clinical trials and translational implementation.

## Data availability

The privacy-protected data is available under reasonable request to the corresponding author.

## Author contributions

Study conception: P.M.G, R.R.L, J.G.R.; data collection: P.M.G, R.R.L, F.S.F., E.S.A., F.C.C., R.E.R.; methodology development: P.M.G., R.R.L., A.C.G., J.G.R., ; data curation and visualization: P.M.G., A.C.G., F.S.F., E.S.A., F.C.C., E.L.S., F.S., R.E.R.; analysis: P.M.G., A.C.G., J.G.R.; manuscript preparation and writing: P.M.G., R.R.L., J.G.R.; manuscript supervision: P.M.G., R.R.L., A.C.G., F.S.F., E.S.A., F.C.C., E.L.S., F.S., R.E.R. J.G.R.; project administration: J.G.R.

All the authors read and approved the final manuscript.

## Funding

This work was supported by the Spanish Ministry of Science, Innovation and Universities (MICIU) through grants PID2021-124427OB-I00 and CNS2023-143743, funded by MICIU/AEI/https://doi.org/10.13039/501100011033 and co-financed ERDF/EU and Next-Generation EU/PRTR. Additional funding was provided by the Andalusian Ministry of University, Research and Innovation under grant ProyExcel-01041. FC-C and PM-G received predoctoral fellowships from INiBICA and the University of Cádiz (UCA PIF program), respectively. AJC-G was supported by a postdoctoral fellowship from the University of Cádiz (“Plan CIE Estabiliza” Program). ES-A was supported by the Spanish Ministry of Science and Innovation through a Juan de la Cierva-Training postdoctoral fellowship (FJC2021--046990-I).

## Declaration of competing interest

Raúl Rashid-López and Raúl Espinosa-Rosso have received honoraria for speaking engagement from Teva, Abbvie, Zambon, BIAL, Lundbeck and Italfarmaco. Javier J. González-Rosa have also received honoraria for speaking engagement from Lundbeck and travel support and training from Medtronic. Paloma Macías-García, Álvaro J- Cruz-Gómez, Fátima Cano-Cano, F-Luis Sánchez-Fernández, Esteban Sarrias-Arrabal, Elena Lozano-Soto, and Florencia Sanmartino declare that they had no comercial or financial relationships that could be construed as potential conflicts of interest during the conduct of this research.

## References

[1] Jankovic J. (2008 Apr 1). Parkinson's disease: clinical features and diagnosis. J Neurol Neurosurg Psychiatry.

[2] Schneider S.A., Obeso J.A. (2014). Clinical and pathological features of parkinson's disease.

[3] Kalia L.V., Lang A.E. (2015 Aug). Parkinson's disease. Lancet.

[4] Poewe W. (2008 Apr 17). Non-motor symptoms in Parkinson's disease. Eur J Neurol.

[5] Schapira A.H.V., Chaudhuri K.R., Jenner P. (2017 Jul 8). Non-motor features of Parkinson disease. Nat Rev Neurosci.

[6] Hacker C.D., Perlmutter J.S., Criswell S.R., Ances B.M., Snyder A.Z. (2012 Dec). Resting state functional connectivity of the striatum in Parkinson's disease. Brain.

[7] Lindenbach D., Bishop C. (2013 Dec). Critical involvement of the motor cortex in the pathophysiology and treatment of Parkinson's disease. Neurosci Biobehav Rev.

[8] Benninger D.H., Hallett M. (2015 Aug 22). Non-invasive brain stimulation for Parkinson's disease: current concepts and outlook 2015. NeuroRehabilitation.

[9] Chou Y hui, Hickey P.T., Sundman M., Song A.W., Chen N kuei (2015 Apr 1). Effects of repetitive transcranial magnetic stimulation on motor symptoms in parkinson disease. JAMA Neurol.

[10] Zeljkovic Jovanovic M., Stanojevic J., Stevanovic I., Ninkovic M., Nedeljkovic N., Dragic M. (2024 Feb 8). Sustained systemic antioxidative effects of intermittent theta burst stimulation beyond neurodegeneration: implications in therapy in 6-Hydroxydopamine model of parkinson's disease. Antioxidants.

[11] Cheng B., Zhu T., Zhao W., Sun L., Shen Y., Xiao W. (2022 Jan 12). Effect of theta burst stimulation-patterned rTMS on motor and nonmotor dysfunction of Parkinson's disease: a systematic review and metaanalysis. Front Neurol.

[12] Rashid-López R., Macías-García P., Cruz-Gómez Á.J., Sánchez-Fernández F.L., Cano-Cano F., Sanmartino F. (2024 Jun). Bilateral primary motor area intermittent theta-burst stimulation may alleviate gait and postural disturbances in Parkinson's disease patients by astrocytic modulation, caudate volume changes, and increased functional neuroplasticity. Parkinsonism Relat Disord.

[13] Fregni F., Boggio P.S., Nitsche M.A., Rigonatti S.P., Pascual-Leone A. (2006). Cognitive effects of repeated sessions of transcranial direct current stimulation in patients with depression. Depress Anxiety.

[14] Panda R., Deluisi J.A., Lee T.G., Davis S., Muñoz-Orozco I., Albin R.L. (2024 Aug). Improving efficacy of repetitive transcranial magnetic stimulation for treatment of Parkinson disease gait disorders. Front Hum Neurosci.

[15] Siebner H.R., Funke K., Aberra A.S., Antal A., Bestmann S., Chen R. (2022 Aug). Transcranial magnetic stimulation of the brain: what is stimulated? – a consensus and critical position paper. Clin Neurophysiol.

[16] Bhat P., Goyal V., Kumaran S.S., Srivastava A.K., Behari M., Dwivedi S.N. (2023 May 1). Mechanisms of 1 Hz inhibitory and 5 Hz excitatory repetitive transcranial magnetic stimulations in Parkinson's disease: a functional magnetic resonance imaging Study. Brain Connect.

[17] Beynel L., Powers J.P., Appelbaum L.G. (2020 May). Effects of repetitive transcranial magnetic stimulation on resting-state connectivity: a systematic review. Neuroimage.

[18] Kirkovski M., Donaldson P.H., Do M., Speranza B.E., Albein-Urios N., Lindsay (2023).

[19] Fox M.D., Buckner R.L., Liu H., Chakravarty M.M., Lozano A.M., Pascual-Leone A. (2014 Oct 14). Resting-state networks link invasive and noninvasive brain stimulation across diverse psychiatric and neurological diseases. Proc Natl Acad Sci.

[20] Fricke C., Duesmann C., Woost T.B., von Hofen-Hohloch J., Rumpf J.J., Weise D. (2019 Mar 7). Dual-Site transcranial magnetic stimulation for the treatment of Parkinson's disease. Front Neurol.

[21] Owens-Walton C., Jakabek D., Power B.D., Walterfang M., Velakoulis D., van Westen D. (2019 Sep 4). Increased functional connectivity of thalamic subdivisions in patients with Parkinson's disease. PLoS One.

[22] Joel D., Weiner I. (1997 Feb). The connections of the primate subthalamic nucleus: indirect pathways and the open-interconnected scheme of basal ganglia-thalamocortical circuitry. Brain Res Rev.

[23] Leite J., Carvalho S., Battistella L.R., Caumo W., Fregni F. (2017 May 23). Editorial: the role of primary motor cortex as a marker and modulator of pain control and emotional-affective processing. Front Hum Neurosci.

[24] Li S., Jiao R., Zhou X., Chen S. (2020 May). Motor recovery and antidepressant effects of repetitive transcranial magnetic stimulation on Parkinson disease. Medicine.

[25] Zhang W., Deng B., Xie F., Zhou H., Guo J.F., Jiang H. (2022 Oct). Efficacy of repetitive transcranial magnetic stimulation in Parkinson's disease: a systematic review and meta-analysis of randomised controlled trials. eClinicalMedicine.

[26] Rashid-López R., Macías-García P., Sánchez-Fernández F.L., Cano-Cano F., Sarrias-Arrabal E., Sanmartino F. (2023 Oct 5). Neuroimaging and serum biomarkers of neurodegeneration and neuroplasticity in Parkinson's disease patients treated by intermittent theta-burst stimulation over the bilateral primary motor area: a randomized, double-blind, sham-controlled, crossover trial study. Front Aging Neurosci.

[27] Gibb W.R.G., Lees A.J. (1988 Sep). A comparison of clinical and pathological features of young- and old-onset Parkinson's disease. Neurology.

[28] Hughes A.J., Daniel S.E., Kilford L., Lees A.J. (1992 Mar 1). Accuracy of clinical diagnosis of idiopathic Parkinson's disease: a clinico-pathological study of 100 cases. J Neurol Neurosurg Psychiatry.

[29] Groppa S., Oliviero A., Eisen A., Quartarone A., Cohen L.G., Mall V. (2012 May). A practical guide to diagnostic transcranial magnetic stimulation: report of an IFCN committee. Clin Neurophysiol.

[30] Mahieux F., Michelet D., Manifacier M.J., Boller F., Fermanian J., Guillard A. (1995). Mini-Mental Parkinson: first validation study of a new bedside test constructed for Parkinson's disease. Behav Neurol.

[31] Dubois B., Slachevsky A., Litvan I., Pillon B. (2000 Dec 12). The FAB: a frontal assessment battery at bedside. Neurology.

[32] Amieva H., Gaestel Y., Dartigues J.F. (2006 Nov 22). The multiple-choice formats (forms F and G) of the benton visual retention test as a tool to detect age-related memory changes in population-based studies and clinical settings. Nat Protoc.

[33] Le Carret N., Rainville C., Lechevallier N., Lafont S., Letenneur L., Fabrigoule C. (2003 Nov). Influence of education on the benton visual retention test performance as mediated by a strategic search component. Brain Cogn.

[34] Hamilton M. (1960 Feb 1). A rating scale for depression. J Neurol Neurosurg Psychiatry.

[35] Hamilton M. (1959 Mar). The assessment of anxiety states by rating. Br J Med Psychol.

[36] Starkstein S.E. (2012 Feb 11). Apathy in Parkinson's disease: diagnostic and etiological dilemmas. Mov Disord.

[37] Moore S.R., Gresham L.S., Bromberg M.B., Kasarkis E.J., Smith R.A. (1997 Jul 1). A self report measure of affective lability. J Neurol Neurosurg Psychiatry.

[38] Brown R.G., Dittner A., Findley L., Wessely S.C. (2005 Jan). The Parkinson fatigue scale. Parkinsonism Relat Disord.

[39] Peto V., Jenkinson C., Fitzpatrick R., Greenhall R. (1995 Jun). The development and validation of a short measure of functioning and well being for individuals with Parkinson's disease. Qual Life Res.

[40] Grace J., Stout J.C., Malloy P.F. (1999 Sep 1). Assessing frontal lobe behavioral syndromes with the frontal lobe personality scale. Assessment.

[41] Martinez-Martin P., Frades-Payo B., Agüera-Ortiz L., Ayuga-Martinez A. (2012 Nov 18). A short scale for evaluation of neuropsychiatric disorders in Parkinson's disease: first psychometric approach. J Neurol.

[42] Brandstaedter D., Spieker S., Ulm G., Siebert U., Eichhorn T.E., Krieg J.C. (2005 Sep 1). Development and evaluation of the Parkinson psychosis questionnaire. J Neurol.

[43] Brunt E.R., Brooks D.J., Korczyn A.D., Montastruc J.L., Stocchi F. (2002 Apr). Accuracy of clinical diagnosis of idiopathic Parkinson's disease: a clinico-pathological study of 100 cases. J Neural Transm.

[44] Im J.H., Ha J.H., Cho I.S., Lee M.C. (2003 Jan 1). Ropinirole as an adjunct to levodopa in the treatment of Parkinson's disease. J Neurol.

[45] Mizuno Y., Nomoto M., Kondo T., Hasegawa K., Murata M., Takeuchi M. (2013 Sep 25). Transdermal rotigotine in early stage Parkinson's disease: a randomized, double-blind, placebo-controlled trial. Mov Disord.

[46] Rush A.J., South C., Jain S., Agha R., Zhang M., Shrestha S. (2021 Jul). Target JNL: neuropsychiatric disease and treatment clinically significant changes in the 17- and 6-Item Hamilton rating scales for depression: a STAR∗D report. Neuropsychiatr Dis Treat.

[47] Hengartner M.P., Plöderl M. (2022 Apr). Estimates of the minimal important difference to evaluate the clinical significance of antidepressants in the acute treatment of moderate-to-severe depression. BMJ Evid Based Med.

[48] Fan J qi, Lu W jing, qiang Tan W., Liu X., Wang Y ting, bu Wang N. (2022 Sep 21). Effectiveness of acupuncture for anxiety among patients with parkinson disease. JAMA Netw Open.

[49] Yang C., Guo Z., Peng H., Xing G., Chen H., McClure M.A. (2018 Nov 28). Repetitive transcranial magnetic stimulation therapy for motor recovery in Parkinson's disease: a meta-analysis. Brain Behav.

[50] Shulman L.M., Gruber-Baldini A.L., Anderson K.E., Fishman P.S., Reich S.G., Weiner W.J. (2010 Jan 1). The clinically important difference on the unified parkinson's disease rating scale. Arch Neurol.

[51] Owens-Walton C., Jakabek D., Power B.D., Walterfang M., Hall S., van Westen D. (2019).

[52] Ferrer-Gallardo V.J., Esteban-Peñalba T., Rodriguez-Oroz M.C., Caballero-Gaudes C., Paz-Alonso P.M. (2025 Sep 30). Thalamic nuclei volume changes associated with cognitive and motor manifestations of Parkinson's disease. NPJ Parkinsons Dis.

[53] Wang S., Zhang Y., Lei J., Guo S. (2021 Jan 18). Investigation of sensorimotor dysfunction in Parkinson disease by resting-state fMRI. Neurosci Lett.

[54] Carrillo F., Palomar F.J., Conde V., Diaz-Corrales F.J., Porcacchia P., Fernández-del-Olmo M. (2013 Jul). Study of cerebello-thalamocortical pathway by transcranial magnetic stimulation in parkinson's disease. Brain Stimul.

[55] Chen Z., He C., Zhang P., Cai X., Huang W., Chen X. (2023 Apr 1). Abnormal cerebellum connectivity patterns related to motor subtypes of Parkinson's disease. J Neural Transm.

[56] Wu T., Hallett M. (2013 Mar 1). The cerebellum in Parkinson's disease. Brain.

[57] Bostan A.C., Dum R.P., Strick P.L. (2013 May). Cerebellar networks with the cerebral cortex and basal ganglia. Trends Cognit Sci.

[58] Grobe-Einsler M., Baljasnikowa V., Faikus A., Schaprian T., Kaut O. (2024 Oct 5). Cerebellar transcranial magnetic stimulation improves motor function in Parkinson's disease. Ann Clin Transl Neurol.

[59] Bostan A.C., Dum R.P., Strick P.L. (2010 May 4). The basal ganglia communicate with the cerebellum. Proc Natl Acad Sci.

[60] Kawabata K., Watanabe H., Bagarinao E., Ohdake R., Hara K., Ogura A. (2020 Nov). Cerebello-basal ganglia connectivity fingerprints related to motor/cognitive performance in Parkinson's disease. Parkinsonism Relat Disord.

[61] Utevsky A.V., Smith D.V., Huettel S.A. (2014 Jan 15). Precuneus is a functional core of the default-mode network. J Neurosci.

[62] Yu H., Sternad D., Corcos D.M., Vaillancourt D.E. (2007 Mar). Role of hyperactive cerebellum and motor cortex in Parkinson's disease. Neuroimage.

[63] Cunningham S.I., Tomasi D., Volkow N.D. (2017 Feb). Structural and functional connectivity of the precuneus and thalamus to the default mode network. Hum Brain Mapp.

[64] Chen Z., He C., Zhang P., Cai X., Li X., Huang W. (2024 Feb 10). Brain network centrality and connectivity are associated with clinical subtypes and disease progression in Parkinson's disease. Brain Imaging Behav.

[65] Halassa M.M., Kastner S. (2017 Dec 28). Thalamic functions in distributed cognitive control. Nat Neurosci.

[66] Thibes R.B., Novaes N.P., Lucato L.T., Campanholo K.R., Melo L.M., Leite C.C. (2017 Dec). Altered functional connectivity between precuneus and motor systems in Parkinson's disease patients. Brain Connect.

[67] Meister I.G., Krings T., Foltys H., Boroojerdi B., Müller M., Töpper R. (2004 May). Playing piano in the mind—an fMRI study on music imagery and performance in pianists. Cogn Brain Res.

[68] Dordevic M., Hoelzer S., Russo A., García Alanis J.C., Müller N.G. (2022 Aug 15). The role of the precuneus in human spatial updating in a real environment setting—A cTBS study.

[69] Sarasso E., Gardoni A., Zenere L., Emedoli D., Balestrino R., Grassi A. (2024 Sep 6). Neural correlates of bradykinesia in Parkinson's disease: a kinematic and functional MRI study. NPJ Parkinsons Dis.

[70] Grafton S.T., Mazziotta J.C., Woods R.P., Phelps M.E. (1992). Human functional anatomy of visually guided finger movements. Brain.

[71] Deiber M.P., Passingham R.E., Colebatch J.G., Friston K.J., Nixon P.D., Frackowiak R.S.J. (1991 Apr). Cortical areas and the selection of movement: a study with positron emission tomography. Exp Brain Res.

[72] Chen L., Huang T., Ma D., Chen Y.C. (2022 Jun 3). Altered default mode network functional connectivity in parkinson's disease: a resting-state functional magnetic resonance imaging study. Front Neurosci.

[73] Tomasi D., Volkow N.D., Wang R., Telang F., Wang G.J., Chang L. (2009 Jun 30). Dopamine transporters in striatum correlate with deactivation in the default mode network during visuospatial attention. PLoS One.

[74] Zhong J., Guan X., Zhong X., Cao F., Gu Q., Guo T. (2019 Jul). Levodopa imparts a normalizing effect on default-mode network connectivity in non-demented Parkinson's disease. Neurosci Lett.

[75] Argaman Y., Granovsky Y., Sprecher E., Sinai A., Yarnitsky D., Weissman-Fogel I. (2022 Apr). Clinical effects of repetitive transcranial magnetic stimulation of the motor cortex are associated with changes in resting-state functional connectivity in patients with fibromyalgia syndrome. J Pain.

[76] Wang Y., Wang L., Ni X., Jiang M., Zhao L. (2024 Mar). Efficacy of repetitive transcranial magnetic stimulation with different application parameters for post-stroke cognitive impairment: a systematic review. Front Neurosci.

[77] Zheng H.B., Liu B., Shen J., Xie F., Ji Q.M., Zhu X.Y. (2022 Dec). Non-invasive brain stimulation for treating psychiatric symptoms in Parkinson's disease: a systematic review and meta-analysis. J Clin Neurosci.

[78] Chou T., Deckersbach T., Dougherty D.D., Hooley J.M. (2023 Jun 12). The default mode network and rumination in individuals at risk for depression. Soc Cogn Affect Neurosci.

[79] Hamilton J.P., Farmer M., Fogelman P., Gotlib I.H. (2015 Aug). Depressive rumination, the default-mode network, and the dark matter of clinical neuroscience. Biol Psychiatry.

[80] Huang P., Guan X., Guo T., Zeng Q., Xuan M., Gu Q. (2020 Mar). Damaged insula network contributes to depression in Parkinson's disease. Front Psychiatr.

[81] Benoit M., Robert P.H. (2011 Nov). Imaging correlates of apathy and depression in Parkinson's disease. J Neurol Sci.

[82] Davey C.G., Harrison B.J., Yücel M., Allen N.B. (2012 Oct 9). Regionally specific alterations in functional connectivity of the anterior cingulate cortex in major depressive disorder. Psychol Med.

[83] Matsui H., Nishinaka K., Oda M., Niikawa H., Komatsu K., Kubori T. (2007 Sep 20). Depression in Parkinson's disease. J Neurol.

[84] Wei L., Hu X., Yuan Y., Liu W., Chen H. (2018 Jul). Abnormal ventral tegmental area-anterior cingulate cortex connectivity in Parkinson's disease with depression. Behav Brain Res.

[85] Owens-Walton C., Jakabek D., Power B.D., Walterfang M., Velakoulis D., van Westen D. (2019 Sep 4). Increased functional connectivity of thalamic subdivisions in patients with Parkinson's disease. PLoS One.

[86] Dan R., Růžička F., Bezdicek O., Růžička E., Roth J., Vymazal J. (2017 Sep 22). Separate neural representations of depression, anxiety and apathy in Parkinson's disease. Sci Rep.

[87] Liao H., Cai S., Shen Q., Fan J., Wang T., Zi Y. (2021 Feb 9). Networks are associated with depression in patients with parkinson's disease: a resting-state imaging study. Front Neurosci.

[88] Hu X., Song X., Yuan Y., Li E., Liu J., Liu W. (2015 Feb 27). Abnormal functional connectivity of the amygdala is associated with depression in Parkinson's disease. Mov Disord.

[89] Li T., Le W., Jankovic J. (2023 Nov 26). Linking the cerebellum to Parkinson disease: an update. Nat Rev Neurol.

[90] Wang H., Chen H., Wu J., Tao L., Pang Y., Gu M. (2018 May). Altered resting-state voxel-level whole-brain functional connectivity in depressed Parkinson's disease. Parkinsonism Relat Disord.

[91] Lee G.P., Meador K.J., Loring D.W., Allison J.D., Brown W.S., Paul L.K. (2004 Mar). Neural substrates of emotion as revealed by functional magnetic resonance imaging. Cognit Behav Neurol.

[92] Stoodley C., Schmahmann J. (2009 Jan 15). Functional topography in the human cerebellum: a meta-analysis of neuroimaging studies. Neuroimage.

[93] Su Q., Yao D., Jiang M., Liu F., Jiang J., Xu C. (2015 Jan 13). Increased functional connectivity strength of right inferior temporal gyrus in first-episode, drug-naive somatization disorder. Aust N Z J Psychiatr.

[94] Zhang P., Zhang Y., Luo Y., Wang L., Wang K. (2022 Dec 16). Regional activity alterations in Parkinson's disease patients with anxiety disorders: a resting-state functional magnetic resonance imaging study. Front Aging Neurosci.

[95] Harrington D.L., Shen Q., Theilmann R.J., Castillo G.N., Litvan I., Filoteo J.V. (2018 Oct 23). Altered functional interactions of inhibition regions in cognitively normal parkinson's disease. Front Aging Neurosci.

[96] Geng H., Li X., Chen J., Li X., Gu R. (2016 Jan). Decreased Intra- and inter-salience network functional connectivity is related to trait anxiety in adolescents. Front Behav Neurosci.

[97] Xiong H., Guo R.J., Shi H.W. (2020 Jan 14). Altered default mode network and salience network functional connectivity in patients with generalized anxiety disorders: an ICA-Based resting-state fMRI study. Evid Based Complement Alternat Med.

[98] Ball T.M., Ramsawh H.J., Campbell-Sills L., Paulus M.P., Stein M.B. (2013 Jul 31). Prefrontal dysfunction during emotion regulation in generalized anxiety and panic disorders. Psychol Med.

[99] Cha J., DeDora D., Nedic S., Ide J., Greenberg T., Hajcak G. (2016 Apr 27). Clinically anxious individuals show disrupted feedback between inferior frontal gyrus and prefrontal-limbic control circuit. J Neurosci.

[100] Adamaszek M., Kirkby K.C. (2022). The neurophysiology of the cerebellum in emotion.

[101] Habas C. (2021 Apr 8). Functional connectivity of the cognitive cerebellum. Front Syst Neurosci.

[102] Guye M., Parker G.J.M., Symms M., Boulby P., Wheeler-Kingshott C.A.M., Salek-Haddadi A. (2003 Aug). Combined functional MRI and tractography to demonstrate the connectivity of the human primary motor cortex in vivo. Neuroimage.

[103] Neggers S.F.W., Zandbelt B.B., Schall M.S., Schall J.D. (2015 Apr). Comparative diffusion tractography of corticostriatal motor pathways reveals differences between humans and macaques. J Neurophysiol.

[104] Vink J.J.T., Mandija S., Petrov P.I., van den Berg C.A.T., Sommer I.E.C., Neggers S.F.W. (2018 Nov 29). A novel concurrent TMS-fMRI method to reveal propagation patterns of prefrontal magnetic brain stimulation. Hum Brain Mapp.

[105] Leodori G., Fabbrini A., De Bartolo M.I., Costanzo M., Asci F., Palma V. (2021 Oct). Cortical mechanisms underlying variability in intermittent theta-burst stimulation-induced plasticity: a TMS-EEG study. Clin Neurophysiol.

[106] Ji G., Liu T., Li Y., Liu P., Sun J., Chen X. (2021 Apr 15). Structural correlates underlying accelerated magnetic stimulation in Parkinson's disease. Hum Brain Mapp.

[107] Vernet M., Bashir S., Yoo W., Perez J.M., Najib U., Pascual-Leone A. (2013 Feb 28). Insights on the neural basis of motor plasticity induced by theta burst stimulation from TMS-EEG. Eur J Neurosci.

[108] Watanabe T., Hanajima R., Shirota Y., Ohminami S., Tsutsumi R., Terao Y. (2014 May 29). Bidirectional effects on interhemispheric resting-state functional connectivity induced by excitatory and inhibitory repetitive transcranial magnetic stimulation. Hum Brain Mapp.

[109] Larson J., Munkácsy E. (2015 Sep). Theta-burst LTP. Brain Res.

[110] Lomarev M.P., Kanchana S., Bara-Jimenez W., Iyer M., Wassermann E.M., Hallett M. (2006 Mar 9). Placebo-controlled study of rTMS for the treatment of Parkinson's disease. Mov Disord.

[111] Hawes S.L., Gillani F., Evans R.C., Benkert E.A., Blackwell K.T. (2013 Nov 1). Sensitivity to theta-burst timing permits LTP in dorsal striatal adult brain slice. J Neurophysiol.

[112] Sharbafshaaer M., Cirillo G., Esposito F., Tedeschi G., Trojsi F. (2024 Nov 1). Harnessing brain plasticity: the therapeutic power of repetitive transcranial magnetic stimulation (rTMS) and Theta Burst Stimulation (TBS) in neurotransmitter modulation, receptor dynamics, and neuroimaging for neurological innovations. Biomedicines.

[113] Strafella A.P. (2005). Proceedings. 2005 IEEE international joint conference on neural networks, 2005.

[114] Rashid-López R., Macías-García P., Cruz-Gómez Á.J., Cano-Cano F., Sánchez-Fernández F.L., Sarrias-Arrabal E. (2026 Feb 6). Plasticity-induced motor recovery of bilateral intermittent theta burst stimulation in Parkinson's disease: a randomized, double-blind, sham-controlled, crossover trial. Neurol Ther.

