# Supplementary material for: Bilateral intermittent theta burst stimulation over the primary motor cortex improves motor and affective symptoms via thalamic network reintegration in mid-stage Parkinson's disease
Source: Neurotherapeutics. 2026 Apr 28;23(3):e00911. doi: 10.1016/j.neurot.2026.e00911 (PMC13141711; doi:10.1016/j.neurot.2026.e00911)
Supplement: Multimedia component 1 [file mmc1.docx]

**Supplementary Material**


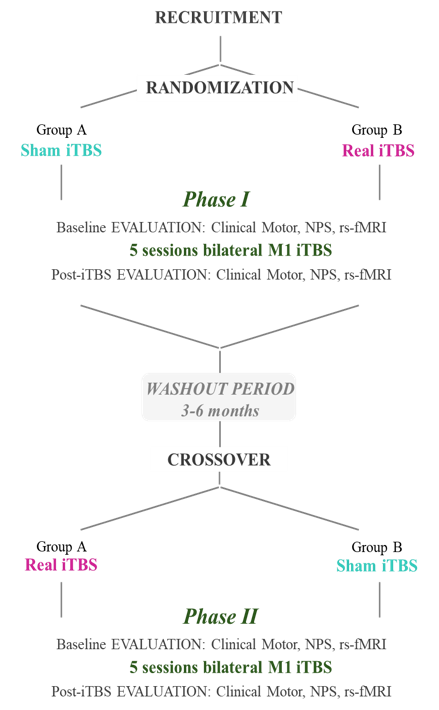


**Supplementary Fig 1.**

Crossover, double-blind, sham controlled study protocol

**fMRI preprocessing methodology**

After the MRI data were acquired, images were converted to NIfTI format and manually aligned to the anterior and posterior commissure (AC & PC) using Statistical Parametric Mapping (SPM12; fil.ion.ucl.ac.uk/spm/). Rs-fMRI data preprocessing was conducted with the Data Processing & Analysis for Brain Imaging tool (DPARSF V4.3 - http://rfmri.org/DPARSF). The preprocessing pipeline included the removal of the first 10 functional volumes, slice-timing correction, realignment to the first volume, head-motion correction, coregistration, nuisance covariate regression, spatial normalization to MNI space, spatial smoothing with a 4-mm FWHM Gaussian kernel, and temporal filtering (0.01 Hz – 0.1 Hz). First-level analyses involved extracting the residual BOLD time series from each seed region and calculating Pearson correlation coefficients with the time series of all other voxels in the brain. These coefficients were Fisher-transformed into z scores to ensure normality, creating seed-to-whole-brain correlation maps. The resulting z score maps were subsequently used for second-level general linear model analyses. For group-level analyses, these maps were processed using tools such as SPM12 and SPSS.

**Supplementary Table 1.** Factorial analysis for MDS-UPDRS

| 1. **MDS-UPDRS part III** | | | | | | |
| --- | --- | --- | --- | --- | --- | --- |
|  | **Treatment** | | **Timepoint** | | **Treatment * Timepoint** | |
|  | *F* | *p* | *F* | *p* | *F* | *p* |
| **MDS-UPDRS part II** | 0.183 | 0.675 | 2.522 | 0.095 | 1.361 | 0.272 |
| **MDS-UPDRS part III** | 0.210 | 0.654 | 14.883 | **< 0.001 ^a^** | 5.235 | **0.014 ^a^** |
| **MDS-UPDRS part IV** | 0.068 | 0.798 | 6.532 | **0.010 ^a^** | 0.757 | 0.433 |
|  |  |  |  |  |  |  |
| 1. **MDS-UPDRS part III clinical symptoms subdomains** | | | | | | |
|  | **Treatment** | | **Timepoint** | | **Treatment * Timepoint** | |
|  | *F* | *p* | *F* | *p* | *F* | *p* |
| **Bradykinesia** | 0.066 | 0.801 | 5.836 | **0.001 ^a^** | 3.809 | **0.008 ^a^** |
| **Tremor** | 0.054 | 0.819 | 3.842 | **0.008 ^a^** | 2.884 | **0.031 ^a^** |
| **Rigidity** | 0.000 | 1.000 | 9.494 | **< 0.001 ^a^** | 2.021 | 0.136 |
| **Axial** | 0.174 | 0.683 | 4.261 | **0.004 ^a^** | 0.093 | 0.984 |

**Table A:** MDS-UPDRS part II, part III and part IV two-way ANOVA for repeated measures results, with treatment (iTBS real, iTBS sham) and time point (baseline, post-1, post-2, post-3, post-4) as within-subject factors. **Table B:** MDS-UPDRS part III clinical symptoms subdomains (bradykinesia, tremor, rigidity and axial symptomatology) two-way ANOVA for repeated measures results, with treatment (iTBS real, iTBS sham) and time point (baseline, post-1, post-2, post-3, post-4) as within-subject factors. Statistical significance was set at p ≤0.05. (**a**) indicates statistical significance to baseline values; (**b**) indicates a trend towards significance to baseline values. **Abbreviations**: MDS-UPDRS, Movement Disorder Society-Unified Parkinson’s Disease Rating Scale, Parts II, III, and IV.

**Supplementary Table 2.** Factorial analysis for cognitive and neuropsychiatric outcomes

|  | | **Treatment** | | **Timepoint** | | | **Treatment * Timepoint** | | |
| --- | --- | --- | --- | --- | --- | --- | --- | --- | --- |
|  | ***F*** | | ***p*** | | ***F*** | ***p*** | | ***F*** | ***p*** |
| **MMP** | 2.513 | | 0.135 | | 1.119 | 0.308 | | 4.293 | 0.057 **^b^** |
| **FAB** | 1.668 | | 0.217 | | 0.023 | 0.882 | | 0.344 | 0.567 |
| **BVRB** | 1.857 | | 0.194 | | 0.169 | 0.687 | | 1.021 | 0.329 |
| **PFS-16** | 0.345 | | 0.566 | | 3.380 | 0.087 **^b^** | | 0.417 | 0.529 |
| **HDRS** | 0.296 | | 0.595 | | 10.499 | **0.006 ^a^** | | 0.144 | 0.710 |
| **HARS** | 0.189 | | 0.670 | | 13.180 | **0.003 ^a^** | | 0.976 | 0.340 |
| **CNS-LS** | 0.937 | | 0.349 | | 3.958 | 0.067 **^b^** | | 0.258 | 0.619 |
| **FrSBE- ED** | 0.120 | | 0.734 | | 0.131 | 0.723 | | 0.593 | 0.454 |
| **FrSBE- APATHY** | 3.278 | | 0.092**^b^** | | 0.776 | 0.393 | | 0.123 | 0.731 |
| **FrSBE-DIS** | 0.816 | | 0.382 | | 2.858 | 0.113 | | 1.892 | 0.191 |
| **FrSBE-TOTAL** | 0.908 | | 0.357 | | 2.466 | 0.139 | | 1.077 | 0.317 |
| **SAS** | 0.075 | | 0.788 | | 2.099 | 0.169 | | 0.024 | 0.879 |
| **PDQ-MOB** | 0.070 | | 0.796 | | 3.295 | 0.091 **^b^** | | 0.938 | 0.349 |
| **PDQ-DAL** | 0.077 | | 0.785 | | 1.270 | 0.279 | | 3.511 | 0.082 **^b^** |
| **PDQ-EMO** | 0.428 | | 0.523 | | 1.235 | 0.285 | | 1.571 | 0.231 |
| **PDQ-STIG** | 3.116 | | 0.099 **^b^** | | 0.318 | 0.582 | | 1.074 | 0.318 |
| **PDQ-SUPPORT** | 0.398 | | 0.539 | | 0.921 | 0.353 | | 1.778 | 0.204 |
| **PDQ-COGN** | 0.273 | | 0.609 | | 0.335 | 0.572 | | 0.292 | 0.598 |
| **PDQ-COMUN** | 1.811 | | 0.200 | | 0.165 | 0.691 | | 0.001 | 0.979 |
| **PDQ-PAIN** | 0.870 | | 0.367 | | 2.948 | 0.108 | | 1.592 | 0.228 |
| **PDQ-TOTAL** | 0.625 | | 0.442 | | 1.323 | 0.269 | | 0.001 | 0.976 |
| **SEND-PSYC** | 0.000 | | 1.000 | | 0.384 | 0.546 | | 1.672 | 0.217 |
| **SEND-APATHY** | 0.510 | | 0.487 | | 1.000 | 0.334 | | 1.777 | 0.204 |
| **SEND-ICD** | 1.000 | | 0.334 | | 1.000 | 0.334 | | 1.000 | 0.334 |
| **SEND-TOTAL** | 1.069 | | 0.319 | | 1.604 | 0.226 | | 0.145 | 0.709 |
| **PPQ-SLEEP** | 0.037 | | 0.851 | | 5.527 | **0.034 ^a^** | | 0.332 | 0.573 |
| **PPQ-HALL** | 1.000 | | 0.334 | | N.A. | N.A. | | N.A. | N.A. |
| **PPQ-DEL** | N.A. | | N.A. | | 1.000 | 0.334 | | N.A. | N.A. |
| **PPQ-DISOR** | 0.882 | | 0.364 | | 1.424 | 0.253 | | 0.528 | 0.479 |
| **PPQ-TOTAL** | 0.112 | | 0.742 | | 3.744 | 0.073 **^b^** | | 1.620 | 0.224 |

**Note.** Two-way ANOVA for repeated measures results, with treatment (iTBS real, iTBS sham) and time point (baseline, post) as within-subject factors. Statistical significance was set at p ≤0.05. (**a**) indicates statistical significance; (**b**) indicates a trend towards significance. **Abbreviations:** BVRB, Benton Visual Rating Battery; CNS-LS, Center for Neurologic Study-lability Scale; FAB, Frontal Assessment Battery; FrSBE-ED, Frontal Systems Behavior Scale – Executive Dysfunction domain; FrSBE-APATHY, Frontal Systems Behavior Scale – Apathy domain; FrSBE-DIS,Frontal Systems Behavior Scale – Disinhibition; FrSBE-TOTAL, Frontal Systems Behavior Scale – Total score; HARS, Hamilton Anxiety Rating Scale; HDRS, Hamilton Depression Rating Scale; MMP, Mini Mental Parkinson; SAS, Starkstein apathy scale; PDQ-MOB, Parkinson’s Disease Questionnaire – Mobility domain; PDQ-DAL, Parkinson’s Disease Questionnaire – Activities of daily living domain; PDQ-EMO, Parkinson’s Disease Questionnaire – Emotional wellbeing domain; PDQ-STIG, Parkinson’s Disease Questionnaire – Social stigma domain; PDQ-SUPPORT, Parkinson’s Disease Questionnaire – Social support domain; PDQ-COGN, Parkinson’s Disease Questionnaire – Cognition domain; PDQ-COMMUN, Parkinson’s Disease Questionnaire – Communication domain; PDQ-PAIN, Parkinson’s Disease Questionnaire – Pain domain; PDQ-TOTAL, Parkinson’s Disease Questionnaire – Total score; PFS-16, Parkinson’s Fatigue Scale; PPQ-SLEEP, Parkinson’s Psychosis Questionnaire – Sleep disturbances domain; PPQ-HALL, Parkinson’s Psychosis Questionnaire – Hallucinations domain; PPQ-DEL, Parkinson’s Psychosis Questionnaire – Delusions domain; PPQ-DISOR, Parkinson’s Psychosis Questionnaire – Spatiotemporal disorientation domain: PPQ-TOTAL, Parkinson’s Psychosis Questionnaire – Total Score; SEND-PSYCH, Scale for the evaluation of neuropsychiatric disorders in Parkinson’s disease – Psychotic symptomatology domain; SEND-APATHY, Scale for the evaluation of neuropsychiatric disorders in Parkinson’s disease – Apathetic symptomatology domain; SEND-ICD, Scale for the evaluation of neuropsychiatric disorders in Parkinson’s disease – Impulse Control Disorder symptomatology domain; SEND-TOTAL, Scale for the evaluation of neuropsychiatric disorders in Parkinson’s disease – Total Score.

**Supplementary Table 3.** Cognitive and neuropsychiatric tests direct score for iTBS sham and iTBS real

|  | **Baseline iTBS sham** | **Post-iTBS sham** | **t value** | ***p-value*** | **Baseline iTBS real** | **Post iTBS real** | **t value** | ***p-value*** | **t value** | **Δ iTBS sham *vs* Δ iTBS real**  ***p-value*** |
| --- | --- | --- | --- | --- | --- | --- | --- | --- | --- | --- |
| **MMP** | 29.3 ± 2.9 | 28.6 ± 2.8 | 1.195 | 0.252 | 27.9 ± 2.7 | 29.3 ± 3.2 | -2.143 | **0.050^a^** | -2.072 | 0.057 **^b^** |
| **FAB** | 15.3 ± 3.2 | 15.5 ± 3.0 | -0.381 | 0.709 | 16.0 ± 2.2 | 15.9 ± 2.2 | 0.367 | 0.719 | 0.587 | 0.567 |
| **BVRB** | 25.1 ± 5.5 | 24.5 ± 6.5 | 0.517 | 0.613 | 23.3 ± 6.3 | 24.5 ± 5.0 | -0.872 | 0.398 | -1.010 | 0.329 |
| **PFS-16** | 47.3 ± 23.3 | 41.3 ± 17.2 | 1.519 | 0.151 | 42.9 ± 20.2 | 39.7 ± 17.0 | 1.273 | 0.224 | -0.646 | 0.529 |
| **HDRS** | 5.8 ± 7.3 | 3.6 ± 5.2 | 1.884 | 0.081**^b^** | 5.6 ± 4.8 | 2.9 ± 3.3 | 3.696 | **0.002 ^a^** | 0.379 | 0.710 |
| **HARS** | 6.8 ± 9.4 | 4.6 ± 6.7 | 2.048 | 0.060 **^b^** | 7.1 ± 7.0 | 3.3 ± 3.5 | 3.172 | **0.007 ^a^** | 0.988 | 0.340 |
| **CNS-LS** | 12.1 ± 5.0 | 11.3 ± 4.1 | 0.964 | 0.351 | 13.3 ± 5.8 | 11.7 ± 4.6 | 1.737 | 0.104 | 0.508 | 0.619 |
| **FrSBE- ED** | 20.2 ± 9.1 | 20.7 ± 9.1 | -0.308 | 0.762 | 21.9 ± 8.4 | 20.6 ± 8.9 | 0.861 | 0.404 | 0.770 | 0.454 |
| **FrSBE- APATHY** | 18.4 ± 6.0 | 18.1 ± 7.1 | 0.163 | 0.873 | 21.3 ± 6.0 | 20.2 ± 4.7 | 1.031 | 0.320 | 0.350 | 0.731 |
| **FrSBE-DIS** | 17.0 ± 5.9 | 16.5 ± 6.5 | 0.399 | 0.696 | 19.1 ± 5.6 | 16.6 ± 6.0 | 2.651 | **0.019 ^a^** | 1.375 | 0.191 |
| **FrSBE-TOTAL** | 55.6 ± 18.9 | 55.3 ± 20.6 | 0.075 | 0.942 | 62.3 ± 17.1 | 57.4 ± 16.9 | 2.926 | **0.011 ^a^** | 1.038 | 0.317 |
| **SAS** | 9.1 ± 4.7 | 10.3 ± 6.3 | -1.222 | 0.242 | 9.1 ± 3.7 | 10.6 ± 5.5 | -0.987 | 0.340 | -0.155 | 0.879 |
| **PDQ-MOB** | 22.4 ± 18.3 | 21.0 ± 22.6 | 0.411 | 0.688 | 24.0 ± 24.1 | 18.2 ± 17.5 | 2.141 | **0.050 ^a^** | 0.964 | 0.351 |
| **PDQ-DAL** | 19.8 ± 17.1 | 21.6 ± 15.7 | -0.725 | 0.481 | 25.0 ± 18.0 | 18.6 ± 13.3 | 1.893 | 0.079 **^b^** | 1.864 | 0.083 **^b^** |
| **PDQ-EMO** | 27.7 ± 26.8 | 22.0 ± 20.0 | 1.411 | 0.180 | 27.2 ± 22.5 | 26.9 ± 24.6 | 0.102 | 0.920 | -1.247 | 0.233 |
| **PDQ-STIG** | 11.7 ± 16.5 | 5.8 ± 8.0 | 1.768 | 0.099 **^b^** | 12.5 ± 12.3 | 15.0 ± 22.8 | -0.395 | 0.699 | -1.034 | 0.318 |
| **PDQ-SUPPORT** | 5.6 ± 10.8 | 14.1 ± 24.6 | -1.400 | 0.183 | 8.9 ± 15.6 | 6.7 ± 10.1 | 0.541 | 0.597 | 1.327 | 0.206 |
| **PDQ-COGN** | 20.2 ± 21.9 | 16.3 ± 20.1 | 1.291 | 0.218 | 16.7 ± 23.7 | 16.7 ± 19.6 | 0.000 | 1.000 | -0.535 | 0.601 |
| **PDQ-COMUN** | 9.4 ± 12.5 | 10.7 ± 15.5 | -0.560 | 0.584 | 15.6 ± 20.9 | 16.7 ± 24.8 | -0.195 | 0.848 | 0.021 | 0.984 |
| **PDQ-PAIN** | 29.8 ± 30.7 | 23.1 ± 22.7 | 1.808 | 0.092 **^b^** | 31.1 ± 24.9 | 28.3 ± 25.5 | 1.098 | 0.291 | -1.276 | 0.223 |
| **PDQ-TOTAL** | 20.1 ± 14.3 | 18.5 ± 14.8 | 0.862 | 0.403 | 21.1 ± 15.6 | 19.6 ± 13.5 | 1.066 | 0.304 | 0.037 | 0.971 |
| **SEND-PSYC** | 0.3 ± 1.0 | 0.1 ± 0.3 | 1.000 | 0.334 | 0.1 ± 0.5 | 0.2 ± 0.6 | -1.000 | 0.334 | -1.293 | 0.217 |
| **SEND-APATHY** | 1.9 ± 3.1 | 1.7 ± 2.7 | 0.343 | 0.737 | 2.3 ± 3.7 | 1.7 ± 3.5 | 1.418 | 0.178 | 1.333 | 0.204 |
| **SEND-ICD** | 0.0 ± 0.0 | 0.0 ± 0.0 | N.A. | N.A. | 0.1 ± 0.5 | 0.1 ± 0.3 | 1.000 | 0.334 | 1.000 | 0.334 |
| **SEND-TOTAL** | 2.2 ± 3.9 | 1.7 ± 2.9 | 1.023 | 0.324 | 2.6 ± 4.5 | 2.0 ± 3.9 | 1.317 | 0.209 | 0.381 | 0.709 |
| **PPQ-SLEEP** | 2.1 ± 1.8 | 1.2 ± 1.3 | 3.166 | **0.007 ^a^** | 2.0 ± 1.2 | 1.4 ± 1.4 | 1.260 | 0.228 | -0.417 | 0.683 |
| **PPQ-HALL** | 0.0 ± 0.0 | 0.0 ± 0.0 | N.A. | N.A. | 0.1 ± 0.3 | 0.1 ± 0.3 | N.A. | N.A. | N.A. | N.A. |
| **PPQ-DEL** | 0.1 ± 0.3 | 0.1 ± 0.5 | -1.000 | 0.334 | 0.1 ± 0.3 | 0.1 ± 0.5 | -1.000 | 0.334 | N.A. | N.A. |
| **PPQ-DISOR** | 0.1 ± 0.5 | 0.1 ± 0.5 | 0.000 | 1.000 | 0.5 ± 1.6 | 0.1 ± 0.3 | 0.972 | 0.348 | 0.598 | 0.560 |
| **PPQ-TOTAL** | 2.0 ± 2.2 | 1.7 ± 1.9 | 0.638 | 0.534 | 2.6 ± 2.4 | 1.3 ± 1.7 | 2.179 | **0.047 ^a^** | 1.429 | 0.175 |

Neuropsychiatric scores before and after sham iTBS and real iTBS. Data are shown as mean ± sd. Statistical significance was set at p ≤0.05. **(a)** indicates statistical significance; **(b)** indicates a trend towards significance. **Abbreviations***:* BVRB, Benton Visual Rating Battery; CNS-LS, Center for Neurologic Study-lability Scale; FAB, Frontal Assessment Battery; FrSBE-ED, Frontal Systems Behavior Scale – Executive Dysfunction domain; FrSBE-APATHY, Frontal Systems Behavior Scale – Apathy domain; FrSBE-DIS,Frontal Systems Behavior Scale – Disinhibition; FrSBE-TOTAL, Frontal Systems Behavior Scale – Total score; HARS, Hamilton Anxiety Rating Scale; HDRS, Hamilton Depression Rating Scale; MMP, Mini Mental Parkinson; SAS, Starkstein apathy scale; PDQ-MOB, Parkinson’s Disease Questionnaire – Mobility domain; PDQ-DAL, Parkinson’s Disease Questionnaire – Activities of daily living domain; PDQ-EMO, Parkinson’s Disease Questionnaire – Emotional wellbeing domain; PDQ-STIG, Parkinson’s Disease Questionnaire – Social stigma domain; PDQ-SUPPORT, Parkinson’s Disease Questionnaire – Social support domain; PDQ-COGN, Parkinson’s Disease Questionnaire – Cognition domain; PDQ-COMMUN, Parkinson’s Disease Questionnaire – Communication domain; PDQ-PAIN, Parkinson’s Disease Questionnaire – Pain domain; PDQ-TOTAL, Parkinson’s Disease Questionnaire – Total score; PFS-16, Parkinson’s Fatigue Scale; PPQ-SLEEP, Parkinson’s Psychosis Questionnaire – Sleep disturbances domain; PPQ-HALL, Parkinson’s Psychosis Questionnaire – Hallucinations domain; PPQ-DEL, Parkinson’s Psychosis Questionnaire – Delusions domain; PPQ-DISOR, Parkinson’s Psychosis Questionnaire – Spatiotemporal disorientation domain: PPQ-TOTAL, Parkinson’s Psychosis Questionnaire – Total Score; SEND-PSYCH, Scale for the evaluation of neuropsychiatric disorders in Parkinson’s disease – Psychotic symptomatology domain; SEND-APATHY, Scale for the evaluation of neuropsychiatric disorders in Parkinson’s disease – Apathetic symptomatology domain; SEND-ICD, Scale for the evaluation of neuropsychiatric disorders in Parkinson’s disease – Impulse Control Disorder symptomatology domain; SEND-TOTAL, Scale for the evaluation of neuropsychiatric disorders in Parkinson’s disease – Total Score.

**Post-hoc analysis for flexible factorial design**

A series of t tests were conducted considering the ROI and a mask with – the previously significant – cluster of interest to further explore changes within and between groups. Significant differences in baseline rs-FC were found between real iTBS and sham iTBS (t=7.00, k=50 voxels, pFWEc≤0.001, [-54, -33, -24]). No significant differences were found in post-iTBS rs-FC between treatment conditions. Within-group analysis revealed that real iTBS significantly elicited an increase in rs-FC (t = 5.01, k = 120 voxels, pFWEc = 0.002, [-39, -24, -30]), whereas it promoted a decrease – although minor – in rs-FC for sham iTBS (t = 4.01, k = 10 voxels, pFWEc = 0.048, [-45, -24, -27]; (t = 4.95, k = 19 voxels, pFWEc = 0.018, [-57, -36, -21]).
